# Synthesis and Evaluation of Marine-Inspired Compounds Result in Hybrids with Antitrypanosomal and Antileishmanial Activities

**DOI:** 10.3390/md21110551

**Published:** 2023-10-24

**Authors:** Diogo Teixeira Carvalho, Melissa Teixeira, Sara Luelmo, Nuno Santarém, Eugénia Pinto, Anabela Cordeiro-da-Silva, Emília Sousa

**Affiliations:** 1 Laboratory of Organic and Pharmaceutical Chemistry, Department of Chemical Sciences, Faculty of Pharmacy, University of Porto, Rua Jorge Viterbo Ferreira 228, 4050-313 Porto, Portugal; up201706634@edu.ff.up.pt; 2 Laboratory of Research in Pharmaceutical Chemistry, Department of Food and Drugs, Faculty of Pharmaceutical Sciences, Federal University of Alfenas, Rua Gabriel Monteiro da Silva 700, Alfenas 37130-001, Brazil; 3 Laboratory of Microbiology, Department of Biological Sciences, Faculty of Pharmacy, University of Porto, Rua de Jorge Viterbo Ferreira 228, 4050-313 Porto, Portugal; santarem@i3s.up.pt (N.S.); epinto@ff.up.pt (E.P.); cordeiro@ff.up.pt (A.C.-d.-S.); 4 Institute for Research and Innovation in Health (i3S), University of Porto, Rua Alfredo Allen 208, 4200-135 Porto, Portugal; sluelmo@i3s.up.pt; 5 Interdisciplinary Centre of Marine and Environmental Research (CIIMAR), Terminal de Cruzeiros do Porto de Leixões, Av. General Norton de Matos s/n, 4450-208 Matosinhos, Portugal

**Keywords:** quinazolinone, eugenol, natural products, hybrid compounds, 1,2,3-triazoles, *Trypanosoma brucei*, *Leishmania infantum*

## Abstract

Natural products are a very rich source for obtaining new compounds with therapeutic potential. In the search for new antiparasitic and antimicrobial agents, molecular hybrids were designed based on the structures of antimicrobial marine quinazolinones and eugenol, a natural phenolic compound. Following reports of the therapeutic potential of quinazolinones and eugenol derivatives, it was expected that the union of these pharmacophores could generate biologically relevant substances. The designed compounds were obtained by classical synthetic procedures and were characterized by routine spectrometric techniques. Nine intermediates and final products were then evaluated in vitro against *Trypanosoma brucei* and *Leishmania infantum*. Antifungal and antibacterial activity were also evaluated. Six compounds (**9b**, **9c**, **9d**, **10b**, **10c**, and **14**) showed mild activity against *T. brucei* with IC_50_ in the range of 11.17–31.68 μM. Additionally, intermediate **9c** showed anti-*Leishmania* activity (IC_50_ 7.54 μM) and was six times less cytotoxic against THP-1 cells. In conclusion, novel derivatives with a simple quinazolinone scaffold showing selectivity against parasites without antibacterial and antifungal activities were disclosed, paving the way for new antitrypanosomal agents.

## 1. Introduction

Parasites are responsible for a high rate of mortality each year and represent a global health burden worldwide, with 11 parasitic infections identified as neglected tropical diseases by the World Health Organization (WHO) [1,2]. Parasitic infections are rapidly spreading and becoming a major cause of chronic diseases due to climate change and environmental pollution, as well as increased resistance to the used drug arsenal [3].

Human African trypanosomiasis (HAT), also known as sleeping sickness, is a parasite-caused neglected disease that greatly affects sub-Saharan Africa, putting about 55 million people at risk (according to 2020 data) [4]. This potentially fatal disease is caused by two *Trypanosoma brucei* species, namely *Trypanosoma brucei gambiense* and *Trypanosoma brucei rhodesiense*, and the parasites are transmitted to the human host by the bite of the tsetse fly [5,6]. WHO, in collaboration with governmental and nongovernmental organizations, has been able to significantly reduce the incidence of HAT to fewer 1000 cases per year, attributable to access to novel drugs [4,6].

Leishmaniasis is a parasitic neglected disease caused by *Leishmania* spp., which are transmitted to mammal hosts by the bite of infected female sandflies. Visceral leishmaniasis is the most severe and life-threatening form and is caused by *Leishmania donovani* on the Asian and African continents and by *Leishmania infantum* in the Mediterranean Basin (Portugal, Spain, Greece, and Italy), the Middle East, Central Asia, and Central/North America [2,7]. Annually, there are reports of up to 1 million new cases although infection by parasites does not always correlate with the development of disease [8].

The research on novel antiparasitic agents has been limited and represents a low interest investment for large pharmaceutical companies due to their incidence occurring mainly in secluded, poor, and disadvantaged populations with limited resources [9]. A large proportion of the approved and new-drug applicants is occupied by natural products or derivatives, furthers suggesting their impact in medicine over the years [10,11].

**Figure 1:**
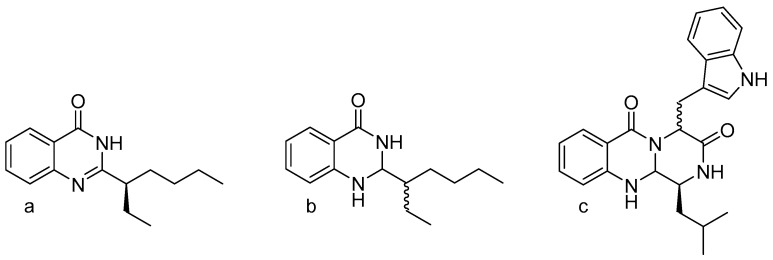
Bioactive quinazolinone (**a**), dihydroquinazolinone (**b**), and indolylmethylpyrazinoquinazoline (**c**) obtained from or inspired by marine sources.

Marine natural products have shown a variety of biological activities, such as antimicrobial, antioxidant, anticancer, and antiparasitic. In terms of antiparasitic activity, various compounds from marine sources have been reported, and reviews of their antiprotozoal potential have shown several examples of novel compounds for this biological activity [9,12]. In particular, quinazolinones have rendered derivatives with several pharmacological and biological activities, such as antibacterial, antifungal, and antiparasitic [13]. Examples of naturally occurring quinazolinones from *Bacillus cereus* present in sea mud with antifungal activity were reported (Figure 1) [14]. Recently, our research group disclosed for the first time new marine-derived indolylmethylpyrazinoquinazolines active against *Plasmodium* and *Trypanosomatids* [2].

**Figure 2:**
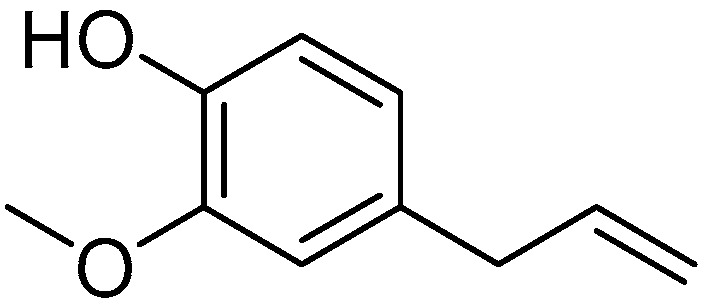
Chemical structure of eugenol.

On the other hand, eugenol, a phenolic natural compound (Figure 2), has presented various biological activities, including antiparasitic and antimicrobial, among others [15,16,17]. Chemical modifications of the eugenol structure or integration of this unit into a given privileged structure is an approach often taken by medicinal chemists to obtain derivatives with improved biological profiles.

Therefore, keeping in mind the diversity and potential of marine compounds, particularly of the marine quinazolinones, a series of derivatives of novel quinazolinones associated with the eugenol moiety was designed via a well-known click chemistry reaction [18]. We hypothesized that this new structure scaffold could be promising against parasitic diseases since it brings together essential moieties from bioactive compounds. The synthesis of these quinazolinone–eugenol and related compounds will be herein discussed, as well as their antiparasitic activity and further screenings for antimicrobial activities.

## 2. Results and Discussion

### 2.1. Chemistry

**Scheme 1:**
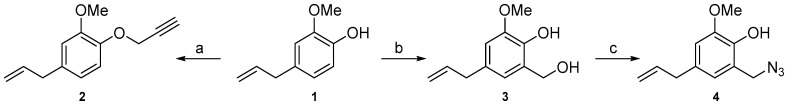
Synthesis of intermediates **2** and **4**: (a) K_2_CO_3_, propargyl bromide, anhydrous DMF, 0 °C to 25 °C, 24 h; (b) NaOH, formaldehyde, H_2_O, 25 °C; (c) SOCl_2_, K_2_CO_3_, anhydrous DMF, 0 °C, 2 h then NaN_3_, anhydrous DMSO, 25 °C, 24 h.

The synthetic route used to obtain the hybrids consisted initially of the functionalization of eugenol (**1**) to obtain the propargyl ether **2** and the azide **4** (see Scheme 1). To obtain **2**, eugenol (**1**) was subjected to *O*-alkylation with propargyl bromide in basic medium, according to a procedure described before [19]. The azide (**4**) was synthesized in three steps, namely by a hydroxymethylation reaction of eugenol (**1**) with formaldehyde in basic medium [20], followed by chlorination of the alcoholic intermediate (**3**) with thionyl chloride and one-pot nucleophilic substitution with sodium azide. Intermediate **4** formation was confirmed by NMR, in which a singlet relative to methylene protons alpha to the azido group could be observed at 4.37 ppm. In the ^13^C NMR spectrum, the signal relative to this group was found in the region expected for this type of carbon at 49.2 ppm.

**Scheme 2:**
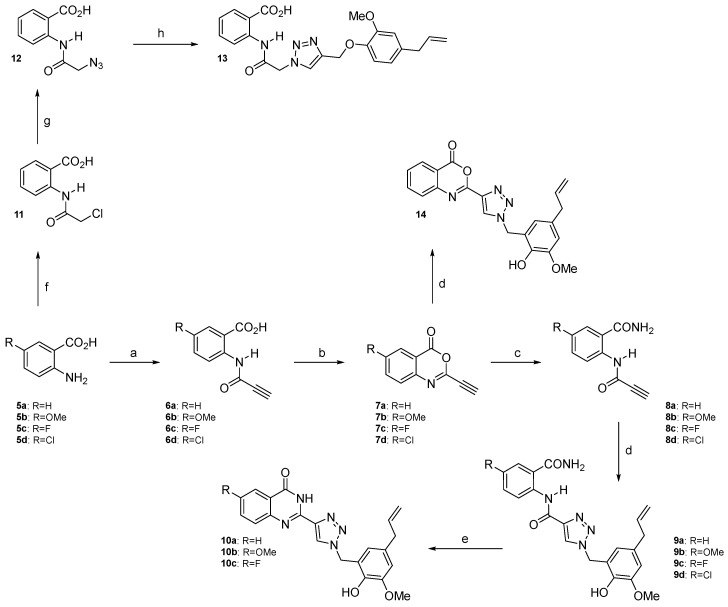
Synthesis of hybrid compounds. (a) Propynoyl chloride, TEA, anhydrous DCM, 0 °C; 1 h; (b) acetic anhydride, 130 °C, 2 h; (c) 28% NH_4_OH, EtOH, 25 °C, 2 h; (d) azide intermediate **4**, CuSO_4_.5H_2_O, sodium ascorbate, THF, H_2_O, 25 °C, 4–24 h; (e) NaOH, H_2_O, EtOH, reflux, 1 h; (f) chloroacetyl chloride, K_2_CO_3_, DCM, 0 °C, 2 h; (g) NaN_3_, DMF, 25 °C, 24 h; (h) propargyl intermediate **2**, CuSO_4_.5H_2_O, sodium ascorbate, THF, H_2_O, 25 °C, 4 h.

The synthetic route used to obtain the quinazolinone counterpart is shown in Scheme 2. First, anthranilic acids **5a**–**5d** were subjected to an *N*-acylation reaction with propynoyl chloride, prepared from propiolic acid and thionyl chloride, as indicated elsewhere [21]. Anthranilic acid (**5a**) was further *N*-acylated with chloroacetyl chloride, and the intermediate **11** was converted into the azide **12**, following the procedures described before [22]. Amide intermediates **6a**–**6d** were treated with acetic anhydride under reflux to form benzoxazinone intermediates **7a**–**7d**, according to the general procedure reported [23]. Our attempts to convert these benzoxazinones directly into the respective quinazolinones by reaction with ammonia did not result in success, contrary to what was reported in another work [24]. In this case, instead of quinazolinones, 2-acylamino-benzamides **8a**–**8d** were obtained as described by Kelleher and coworkers [18], who used this ammonia-based method for the synthesis of this type of benzamide. Intermediates **6a** and **7a** were easy to handle and could be purified and properly characterized. The main findings in the characterization of **6a** by ^1^H NMR were the characteristic signal of the acetylenic proton at 4.51 ppm and the signals for both acetylenic carbons at 79.48 and 78.84 ppm in ^13^C NMR spectra. Still, in the ^13^C NMR spectrum, the signals at 170.71 and 150.84 ppm, relative to the amide and acid carbonyls, established this characterization. For the intermediate benzoxazolone **7a**, the main evidence was the presence of a single carbonyl signal at 159.47 ppm, in addition to the azomethine carbon at 146.79 ppm.

The amides **6b**–**6d**, benzoxazinones **7b**–**7d**, and 2-acylamino-benzamides **8a**–**8d** were found to be quite unstable, decomposing rapidly during purification and characterization attempts. Thus, intermediates **8a**–**8d** were used in crude form for the synthesis of triazole intermediates **9a**–**9d**, while intermediates **12** and **7a** could generate the additional triazoles **13** and **14**, respectively. These and the other triazoles (**9a**–**9d**) were prepared by the copper catalyzed azide–alkyne cycloaddition reaction, following the classical click procedure described elsewhere [25]. The signal relative to the triazole hydrogen of products **9a**–**9d**, **10a**–**10c**, **13**, and **14** was clearly observed in the range of 8.25–8.62 ppm.

Quinazolinone compounds **10a**–**10c** were obtained from **9a**–**9c**, following the method of cyclocondensation of 2-acylamino anthranilamides in hot aqueous NaOH, as described before [26]. Despite our efforts, attempts at cyclization with the chlorinated intermediate (**9d**) were unsuccessful, so it was not possible to evaluate the activity of the respective quinazolinone. The structure characterization was performed by NMR and HRMS techniques (experimental).

### 2.2. Biological Tests

#### 2.2.1. Antiparasitic Activity

The compounds were screened for their antiparasitic activity against *Leishmania infantum* promastigotes and *Trypanosoma brucei* parasites, and the cytotoxicity was also evaluated using THP-1 cells. The results obtained allowed us to calculate the half maximal inhibitory concentration (IC_50_), which is the concentration of compounds that inhibits parasite growth by 50%, and the cytotoxic concentration 50 (CC_50_), which corresponds to the concentration of compounds required to reduce cell viability by 50%. Both measures, IC_50_ and CC_50_, were calculated in µM with a 95% confidence interval and were used to calculate the selectivity index (SI). The antiparasitic activity, cytotoxicity, and SI are depicted in Table 1.

Except for **9c**, the synthesized hybrids presented no detectable antiparasitic activity against *L. infantum* promastigotes until 40 µM (the highest concentration tested). This compound (**9c**) presented an IC_50_ of 7.54 µM (5% CI of 5.85–9.58). Regarding *T. brucei*, six compounds (**9b**, **9c**, **9d**, **10b**, **10c**, and **14**) presented a quantifiable IC_50_. The most potent IC_50_ values were between 20 and 10 µM. Concerning the cytotoxicity against PMA-differentiated THP-1 cells, the tested series did not induce viability loss in the tested concentrations except for **9c**, which presented a CC_50_ value of 45.82 µM (95% CI of 38.04–55.30). All active compounds presented SI values superior to 2.

It is important to note that, among the synthesized compounds, only **9c** presented activity in both parasites. However, it is worth noting that **9c** was also the most toxic molecule from the series, with a CC_50_ value of 45.82 µM. This finding might be suggestive of a non-specific mechanism of action.

The structure–activity relationship revealed that the different substituents used in intermediate **9** and final derivative **10** influenced the antitrypanosomal activity. Compounds **9b** and **9c** (R=OMe and R=F, respectively) presented a quantifiable IC_50_, while **9a** (R=H) did not show any detectable IC_50_. Among derivatives **10**, compounds **10b** (R=OMe) and **10c** (R=F) had similar IC_50_ values to **9b** and **9c**, respectively. The unsubstituted compounds **10a** and **9a** did not show detectable values of IC_50_. It is important to note that **10c** had the most potent IC_50_ value against *T. brucei*, but its IC_50_ value against *L. infantum* promastigote was greater than 40 µM. On the other hand, **9c** was not the most potent anti-trypanosome compound but presented activity against *Leishmania*. This outcome is suggestive of parasite-specific activity. In conclusion, new hybrid compounds were disclosed as promising scaffolds for the synthesis of new antiparasitic molecules.

#### 2.2.2. Antimicrobial Activity

The compounds were additionally evaluated for antimicrobial activity using the broth microdilution method against three different fungal strains and against two different bacteria, and the results were demonstrated as the minimum inhibitory concentration (MIC), which is the minimal concentration to cause inhibition of fungal or bacteria growth according to the Clinical and Laboratory Standards Institute (CLSI) protocols. Voriconazole and gentamicin were used as quality control drugs for the antifungal and antibacterial assays, respectively, and the obtained results were according to the followed norms. The obtained results for the synthesized compounds revealed poor to no antimicrobial activity for the marine eugenol hybrids (Supplementary Material, Table S1).

The tested series presented MIC values of >512 µg/mL against *C. albicans* and *A. fumigatus*, meaning that the synthesized substances are not promising antifungal agents against these strains. The results against *T. rubrum* were slightly improved for compounds **9a**, **9d**, **10a**, **10c**, and **14**, with values ranging from 256 to 512 µg/mL. Nevertheless, the MIC values are still viewed as high; therefore, the compounds were considered not promising, and no further fungal strains were tested.

The MLCs were tested for compounds **9a**, **9d**, **10a**, **10c**, and **14** for *T. rubrum,* and all compounds presented values of >512 µg/mL. For eugenol, the MLC results were between 256 and 512 µg/mL for *T. rubrum* and were >512 µg/mL for *C. albicans* and *A. fumigatus*.

In terms of the antibacterial activity, **9c** demonstrated a minor improvement in MIC values in comparison to the remaining compounds (MIC between 256 and 512 µg/mL, *versus* >512 µg/mL) when tested against *E. coli* and *S. aureus*. For *E. coli* and *S. aureus*, the only MIC evaluated was for **9c**, which presented a value of >512 µg/mL and ≤512 µg/mL, respectively. Once again, the hybrids were not considered promising compounds for this biological activity.

## 3. Materials and Methods

### 3.1. Chemical Procedures

#### 3.1.1. General Methods

Reagents and solvents were purchased from Sigma-Aldrich (St. Louis, MO, USA), Acros Organics (Thermo Fisher Scientific, Geel, Belgium), or Fisher Scientific (Thermo Fisher Scientific, Loughborough, UK) and were used without further purification. Thin layer chromatography (TLC) using Merck silica gel 60 (GF254)-precoated plates (0.2 mm of thickness) with appropriate mobile phases were used to follow reaction progressions. Ultraviolet light (254 and 365 nm) and 3% aqueous FeCl_3_ (for phenolic derivatives) were used to visually detect compounds on chromatograms. When necessary, purifications of the synthesized compounds were performed by flash column chromatography using silica gel 60 (0.040–0.063 mm, Merck, Darmstadt, Germany) or preparative thin layer chromatography (PTLC) using Merck silica gel 60 (GF254) plates. The ^1^H and ^13^C NMR spectra were taken at the Centro de Materiais (CEMUP)–University of Porto on a Bruker Avance III 400 spectrometer (400 MHz for ^1^H and 100 MHz for ^13^C) or at the University of Aveiro on a Bruker Avance 300 spectrometer (300.13 MHz for ^1^H and 75.47 MHz for ^13^C) in DMSO-*d*_6_ or CDCl_3_ (Deutero GmbH, Ely, UK) at room temperature. Chemical shifts are expressed in δ (ppm) values relative to tetramethylsilane (TMS) as an internal reference. Coupling constants are reported in hertz (Hz). ^13^C NMR assignments were made by comparison with the assignments of similar molecules. High-resolution mass spectrometry (HRMS) was performed on an LTQ OrbitrapTM XL hybrid mass spectrometer (Thermo Fischer Scientific, Bremen, Germany) controlled by LTQ Tune Plus 2.5.5 and Xcalibur 2.1.0 in positive mode at CEMUP–University of Porto. The capillary voltage of the electrospray ionization source (ESI) was set to 3.1 kV. The capillary temperature was 275 °C. The sheath gas was at 6 (arbitrary unit, as provided by the software settings). The capillary voltage was 46 V, and the tube lens voltage was 120 V. The synthesis and purification of compounds were undertaken as described in the following sections.

#### 3.1.2. Synthesis and Structure Elucidation

##### Synthesis of 4-allyl-2-methoxy-1-(prop-2-ynyloxy)benzene (**2**), 5-allyl-2-hydroxy-3-methoxybenzyl alcohol (**3**), and 2-[(azidoacetyl)amino]benzoic acid (**12**)

Compounds **2, 3**, and **12** were synthesized and characterized following the works of Irfan et al. (2015) [19], Singh et al. (1998) [20], and Aarjane et al. (2019) [22], respectively. The ^1^H and ^13^C NMR spectra of **2**, **3**, and **12** were in accordance with the reported data.

##### Synthesis of 4-allyl-2-azido-6-methoxyphenol (**4**)

Compound **3** (0.5 g, 2.6 mmol) was dissolved in anhydrous *N*,*N*-dimethylformamide (5.0 mL) and stirring solution cooled by an ice water bath. Then, thionyl chloride (0.62 g, 0.38 mL, 5.2 mmol) was added dropwise to this solution, followed by anhydrous potassium carbonate (1.1 7.8 mmol). The mixture was left under stirring at room temperature for 2 h. Subsequently, sodium azide (0.17 g, 2.6 mmol) was added as a solution in anhydrous dimethylsulfoxide (1.0 mL). The mixture was kept under the same conditions for 24 h when TLC (hexane/ethyl acetate, 7:3) showed the end of the reaction. Water (10 mL) was added to the mixture, and the crude product was extracted with ethyl acetate (5 × 10 mL), the organic phase was dried over anhydrous sodium sulfate, filtered, and evaporated until dry. The pure product was obtained after flash column chromatography (hexane/ethyl acetate, 7:3). 

4-allyl-2-azido-6-methoxyphenol (4): light yellow oil, 35% yield. ^1^H NMR (300 MHz, CDCl_3_) *δ*_H_ 6.68 (s, 2H, H-3 and H-5), 6.04–5.86 (m, 1H, H-8), 5.70 (s, 1H, H-12), 5.14–5.01 (m, 2H, H-9), 4.37 (s, 2H, H-10), 3.89 (s, 3H, H-11), 3.32 (dt, *J* = 5.9, 1.1 Hz, 2H, H-7); ^13^C NMR (75 MHz, CDCl_3_) *δ*_C_ 146.15 (C-6), 141.96 (C-1), 137.18 (C-8), 131.30 (C-4), 121.56 (C-2), 120.68 (C-3), 115.56 (C-9), 110.87 (C-5), 55.78 (C-11), 49.20 (C-10), 39.54 (C-7).

##### Synthesis of Acetylenic Intermediates **6a** and **7a**

Anthranilic acid **5a** (1 eq) and triethylamine (2 eq) were dissolved in dry dichloromethane (10 mL), and the mixture was stirred and cooled to 0 °C in an ice bath. Then, propynoyl chloride (1.2 eq) was added dropwise. The reaction mixture was kept under the same conditions until the consumption of the starting material, which was visualized by TLC (hexane/ethyl acetate, 1:1). Then, the reaction mixture was poured into crushed ice and stirred vigorously to precipitate the product **6a**, which was filtered off under reduced pressure. The product was used in the next step without further purification. Intermediate **6a** (1 eq) was then dissolved in acetic anhydride (20 mL), and the mixture was heated at 130 °C for 2 h. After this time, the reaction mixture was cooled to room temperature and then poured into crushed ice. After vigorous stirring, the solid that precipitated was filtered off under reduced pressure and washed copiously with water. The obtained product (**7a**) was pure enough to be used in the next step.

2-propiolamidobenzoic acid (**6a**): yellow solid, 75% yield. ^1^H NMR (400 MHz, CDCl_3_) *δ*_H_ 11.63 (s, 1H, H-8), 8.31 (d, *J* = 8.4 Hz, 1H, H-6), 8.00 (dd, *J* = 7.9, 1.7 Hz, 1H, H-3), 7.63 (ddd, *J* = 8.4, 7.4, 1.7 Hz, 1H, H-4), 7.24 (td, *J* = 7.6, 1.2 Hz, 1H, H-5), 4.51 (s, 1H, H-12); ^13^C NMR (100 MHz, DMSO-*d*_6_) *δ*_C_ 170.71 (C-7), 150.84 (C-10), 140.68 (C-2), 135.52 (C-4), 132.54 (C-6), 125.37 (C-5), 122.14 (C-3), 118.81 (C-1), 79.48 (C-11), 78.84 (C-12).

2-ethynyl-4*H*-benzo[d][1,3]oxazin-4-one (**7a**): white solid, 90% yield. ^1^H NMR (400 MHz, CDCl_3_) *δ*_H_ 8.14 (ddd, *J* = 7.9, 1.6, 0.7 Hz, 1H, H-6), 8.02–7.88 (m, 1H, H-3), 7.72–7.56 (m, 2H, H-4 and H-5), 4.89 (s, 1H, H-10); ^13^C NMR (100 MHz, DMSO-*d*_6_) *δ*_C_ 159.47 (7), 146.79 (C-8), 143.20 (C-2), 138.27 (C-4), 131.17 (C-6), 129.48 (C-3), 128.29 (C-5), 119.73 (C-1), 83.98 (C-10), 76.80 (C-9).

##### Synthesis of 2-(propioloylamino)benzamides **8a**–**8d**

Compounds **8a**–**8d** were prepared in three steps from the respective anthranilic acids, following the classical methods reported before [18,23]. As the intermediates **6b**–**6d**, **7b**–**7d** and **8a**–**8d** were quite unstable, they were readily used in the subsequent reactions without purification. However, their identities were confirmed indirectly by the success in obtaining the triazole products.

##### Synthesis of Triazoles **9a**–**9d**, **13**, and **14**

The corresponding alkyne (**2** or **8a**–**8d**, 1 eq) and azide (**4** or **12**, 1 eq) intermediates were dissolved in a mixture of tetrahydrofuran-water (9:1), and to this solution was added sodium ascorbate (0.1 eq) and copper II sulfate (0.01 eq). The mixture was left under vigorous magnetic stirring at room temperature for 4–24 h. The progress of the reaction was monitored by TLC (100% ethyl acetate), and once the reaction was complete, the solvent was evaporated using a rotary evaporator, and the resulting solid was pre-purified by liquid-liquid extraction using water and ethyl acetate. The product isolated from the organic phase was purified by crystallization with diethyl ether or flash column chromatography (ethyl acetate/hexane, 9:1), which led to the desired triazoles.

1-(5-allyl-2-hydroxy-3-methoxybenzyl)-*N*-(2-carbamoylphenyl)-1*H*-1,2,3-triazole-4-carboxamide (**9a**): white solid, 65% yield. ^1^H NMR (400 MHz, DMSO-*d*_6_) *δ*_H_ 12.76 (s, 1H, H-9), 9.03 (s, 1H, H-23), 8.63 (d, 1H, *J* = 7.1 Hz, H-3), 8.54 (s, 1H, H-12), 8.27 (s, 1H, H-8), 7.83 (dd, *J* = 7.4, 1.5 Hz, 1H, H-6), 7.71 (s, 1H, H-8´), 7.54 (ddd, *J* = 7.4, 1.5 Hz, 1H, H-4), 7.17 (td, *J* = 7.4, 1.5 Hz, 1H, H-5), 6.81 (s, 1H, H-17), 6.62 (s, 1H, H-19), 5.92 (td, *J* = 16.8, 6.7 Hz, 1H, H-21), 5.58 (s, 2H, H-13), 5.12–4.98 (m, 2H, H-22), 3.77 (s, 3H, H-24), 3.27 (d, *J* = 6.7 Hz, 2H, H-20); ^13^C NMR (100 MHz, DMSO-*d*_6_) *δ*_C_ 170.94 (C-7), 158.68 (C-10), 148.04 (C-16), 143.17 (C-11), 143.07 (C-15), 139.40 (C-2), 138.33 (C-21), 132.61 (C-4), 131.04 (C-18), 129.11 (C-19), 127.76 (C-6), 123.24 (C-12), 122.06 (C-14), 121.68 (C-5), 120.86 (C-1), 120.78 (C-3), 116.07 (C-22), 112.90 (C-17), 56.34 (C-24), 49.45 (C-13), 39.50 (C-20). ESI-HRMS (+) m/z: Anal. Cal. for (C_21_H_21_N_5_O_4_) (M + H)^+^: 408.1672: found: 408.1686.

1-(5-allyl-2-hydroxy-3-methoxybenzyl)-*N*-(2-carbamoyl-4-methoxyphenyl)-1*H*-1,2,3-triazole-4-carboxamide (9b): light yellow solid, 58% yield. ^1^H NMR (400 MHz, DMSO-*d*_6_) *δ*_H_ 12.44 (s, 1H, H-9), 9.03 (s, 1H, H-23), 8.53 (d, *J* = 9.2 Hz, 2H, H-3), 8.49 (s, 1H, H-12), 8.28 (s, 1H, H-8), 7.71 (s, 1H, H-8´), 7.37 (s, 2H, H-6), 7.14 (dd, *J* = 9.2, 3.0 Hz, 1H, H-4), 6.81 (s, 1H, H-17), 6.62 (s, 1H, H-19), 5.97–5.87 (m, 1H, H-21), 5.57 (s, 2H, H-13), 5.09–5.00 (m, 2H, H-22), 3.81 (s, 3H, H-24), 3.79 (s, 3H, H-25), 3.26 (d, *J* = 5.1 Hz, 2H, H-20); ^13^C NMR (100 MHz, DMSO-*d*_6_) *δ*_C_ 170.60 (C-7), 168.89 (C-10), 158.23 (C-5), 154.87 (C-11), 148.04 (C-16), 143.29 (C-15), 138.34 (C-21), 132.57 (C-18), 131.03 (C-2), 127.51 (C-19), 122.38 (C-12), 122.28 (C-3), 122.09 (C-14), 121.67 (C-1), 118.13 (C-4), 116.07 (C-22), 113.99 (C-6), 112.89 (C-17), 56.34 (C-24), 55.97 (C-25), 49.42 (C-13), 39.50 (C-20). ESI-HRMS (+) m/z: Anal. Cal. for (C_22_H_23_N_5_O_5_) (M)^+^: 437.1969: found: 437.1929.

1-(5-allyl-2-hydroxy-3-methoxybenzyl)-*N*-(2-carbamoyl-4-fluorophenyl)-1*H*-1,2,3-triazole-4-carboxamide (**9c**): white solid, 67% yield. ^1^H NMR (400 MHz, DMSO-*d*_6_) *δ*_H_ 12.65 (s, 1H, H-9), 9.03 (s, 1H, H-23), 8.53 (s, 1H, H-12), 7.85 (s, 1H, H-8), 7.73–7.67 (m, 4H, H-3, H-4, H-6 and H-8´), 6.80 (s, 1H, H-17), 6.62 (s, 1H, H-19), 5.97–5.87 (m, 1H, H-21), 5.57 (s, 2H, H-13), 5.08–5.00 (m, 2H, H-22), 3.79 (s, 3H, H-24), 3.26 (d, *J* = 6.7 Hz, 2H, H-20); ^13^C NMR (100 MHz, DMSO-*d*_6_) *δ*_C_ 169.63 (C-7), 158.59 (C-10), 156.70 (d, *J* = 241 Hz, C-5), 149.26 (C-16), 148.04 (C-11), 141.58 (C-15), 138.31 (C-21), 131.04 (C-18), 130.13 (C-2), 129.26 (C-1), 127.77 (C-19), 123.46 (d, *J* = 8 Hz, C-3), 121.70 (C-12), 119.60 (d, *J* = 22 Hz, C-4), 116.06 (C-22), 115.79 (d, *J* = 22 Hz, C-6), 112.89 (C-17), 55.33 (C-24), 49.47 (C-13), 39.49 (C-20). ESI-HRMS (+) m/z: Anal. Cal. for (C_21_H_20_FN_5_O_4_) (M + H)^+^: 426.1578: found: 426.1572.

1-(5-allyl-2-hydroxy-3-methoxybenzyl)-*N*-(2-carbamoyl-4-chlorophenyl)-1*H*-1,2,3-triazole-4-carboxamide (**9d**): white solid, 46% yield. ^1^H NMR (400 MHz, DMSO-*d*_6_) *δ*_H_ 12.72 (s, 1H, H-9), 9.03 (s, 1H, H-23), 8.67 (d, *J* = 9.0 Hz, 1H, H-3), 8.56 (s, 1H, H-12), 8.38 (s, 1H, H-8), 7.91 (d, *J* = 2.5 Hz, 1H, H-6), 7.86 (s, 1H, H-8´), 7.62 (dd, *J* = 9.0, 2.5 Hz, 1H, H-4), 6.80 (d, *J* = 2.0 Hz, 1H, H-17), 6.62 (d, *J* = 2.1 Hz, 1H, H-19), 6.52 (s, 2H, H-13), 5.92 (ddt, *J* = 16.8, 10.0, 6.8 Hz, 1H, H-21), 5.58 (s, 2H, H-13), 5.10–5.00 (m, 2H, H-22), 3.79 (s, 4H, H-24), 3.29–3.16 (m, 2H, H-20); ^13^C NMR (100 MHz, DMSO-*d*_6_) *δ*_C_ 169.61 (C-7), 158.72 (C-10), 148.04 (C-16), 143.08 (C-11), 142.89 (C-15), 138.33 (C-21), 138.31 (C-2), 132.34 (C-4), 131.04 (C-5), 128.76 (C-3), 127.91 (C-19), 127.04 (C-14), 122.45 (C-6), 122.38 (C-1), 121.69 (C-12), 116.07 (C-22), 112.91 (C-17), 56.34 (C-24), 49.48 (C-13), 39.50 (C-20). ESI-HRMS (+) m/z: Anal. Cal. for (C_21_H_20_ClN_5_O_4_) (M + H)^+^: 442.1282: found: 442.1285.

2-(2-(4-((4-allyl-2-methoxyphenoxy)methyl)-1*H*-1,2,3-triazol-1-yl)acetamido) benzoic acid (**13**): light gray solid, 74% yield. ^1^H NMR (400 MHz, DMSO-*d*_6_) *δ*_H_ 12.80 (s, 1H, H-8), 11.27 (s, 1H, H-9), 8.39 (d, *J* = 8.4 Hz, 1H, H-6), 8.26 (s, 1H, H-12), 8.08 (d, *J* = 7.0 Hz, 1H, H-3), 7.60 (t, *J* = 7.0 Hz, 1H, H-4), 7.21 (t, *J* = 7.0 Hz, 1H, H-5), 7.05 (d, *J* = 8.2 Hz, 1H, H-20), 6.80 (d, *J* = 2.1 Hz, 1H, H-17), 6.69 (dd, *J* = 8.1, 2.1 Hz, 1H, H-19), 5.94 (td, *J* = 16.8, 6.7 Hz, 1H, H-22), 5.49 (s, 2H, H-11), 5.12 (s, 2H, H-14), 5.10–4.95 (m, 2H, H-23), 3.73 (s, 3H, H-24), 3.30 (d, *J* = 5.12 Hz, 2H, H-21); ^13^C NMR (100 MHz, DMSO-*d*_6_) *δ*_C_ 172.68 (C-7), 164.76 (C-10), 157.93 (C-16), 148.96 (C-15), 145.70 (C-13), 139.60 (C-2), 139.04 (C-18), 137.81 (C-22), 134.21 (C-6), 133.91 (C-4), 126.07 (C-3), 124.04 (C-5), 123.42 (C-12), 120.05 (C-19), 119.65 (C-1), 115.45 (C-23), 113.87 (C-17), 112.46 (C-20), 74.71 (C-14), 61.78 (C-11), 55.30 (C-24), 38.98 (C-21). ESI-HRMS (+) m/z: Anal. Cal. for (C_22_H_22_N_4_O_5_) (M − H)^+^: 421.1512: found: 421.1548.

2-(1-(5-allyl-2-hydroxy-3-methoxybenzyl)-1*H*-1,2,3-triazol-4-yl)-4*H*-benzo[*d*][1,3]oxazin-4-one (**14**): off-white solid, 81% yield. ^1^H NMR (400 MHz, DMSO-*d*_6_) *δ*_H_ 9.09 (s, 1H, H-23), 8.72 (s, 1H, H-12), 8.15 (dd, *J* = 7.9, 1.5 Hz, 1H, H-6), 7.99–7.90 (m, 1H, H-5), 7.69 (dd, *J* = 7.3, 1.1 Hz, 1H, H-4), 7.62 (td, *J* = 7.6, 1.1 Hz, 1H, H-3), 6.82 (d, *J* = 2.0 Hz, 1H, H-17), 6.68 (d, *J* = 1.9 Hz, 1H, H-19), 5.98–5.88 (m, 1H, H-21), 5.61 (s, 2H, H-13), 5.09–5.00 (m, 2H, H-22), 3.80 (s, 3H, H-24), 3.27 (s, 2H, H-20); ^13^C NMR (100 MHz, DMSO-*d*_6_) *δ*_C_ 158.99 (C-7), 151.56 (C-10), 148.06 (C-16), 146.68 (C-15), 143.17 (C-2), 139.61 (C-18), 138.31 (C-4), 137.38 (C-21), 131.08 (C-11), 129.06 (C-6), 128.61 (C-3), 128.27 (C-5), 127.15 (C-19), 121.91 (C-12), 121.88 (C-14), 117.69 (C-1), 116.11 (C-22), 112.98 (C-17), 56.33 (C-24), 49.52 (C-13), 39.50 (C-20). ESI-HRMS (+) m/z: Anal. Cal. for (C_21_H_18_N_4_O_4_) (M + H)^+^: 391.1406: found: 391.1418.

##### Synthesis of Quinazolinones **10a**–**10c**

A solution of 10 M aqueous NaOH (2.0 mL) was added to compounds **9a**–**9c** (0.2 mmol) solubilized in ethanol (18.0 mL). The mixture was then heated under reflux for 1 h. After that time, the resulting solution was cooled, and the pH was adjusted to 5 with HCl 1 M. The product was obtained by extraction with ethyl acetate, followed by solvent evaporation, needing no further purification.

2-(1-(5-allyl-2-hydroxy-3-methoxybenzyl)-1H-1,2,3-triazol-4-yl)quinazolin-4(3H)-one (**10a**): Light yellow solid. Quantitative yield. ^1^H NMR (400 MHz, DMSO-*d*_6_) *δ*_H_ 12.38 (s, 1H, H-8), 9.09 (s, 1H, H-23), 8.69 (s, 1H, H-12), 8.15 (s, 1H, H-6), 7.82 (s, 1H, H-4), 7.67 (s, 1H, H-3), 7.48 (s, 1H, H-5), 6.81 (s, 1H, H-17), 6.70 (s, 1H, H-19), 5.93 (d, *J* = 6.9 Hz, 1H, H-21), 5.65 (s, 2H, H-13), 5.16–4.97 (m, 2H, H-22), 3.72 (s, 3H, H-24), 3.23 (m, 2H, H-20); ^13^C NMR (100 MHz, DMSO-*d*_6_) *δ*_C_ 162.62 (C-7), 150.01 (C-16), 148.95 (C-10), 147.15 (C-15), 144.15 (C-2), 141.79 (C-18), 139.19 (C-21), 135.96 (C-4), 131.94 (C-14), 128.48 (C-3), 127.96 (C-6), 127.37 (C-19), 122.92 (C-5), 122.69 (C-1), 117.02 (C-22), 113.87 (C-17), 57.21 (C-24), 50.44 (C-13), 40.40 (C-20). ESI-HRMS (+) m/z: Anal. Cal. for (C_21_H_19_N_5_O_3_) (M + H)^+^: 390.1566: found: 390.1564.

2-(1-(5-allyl-2-hydroxy-3-methoxybenzyl)-1*H*-1,2,3-triazol-4-yl)-6-methoxyquinazolin-4(3*H*)-one (**10b**): White solid. Quantitative yield. ^1^H NMR (400 MHz, DMSO-*d*_6_) *δ*_H_ 12.38 (s, 1H, H-8), 9.03 (s, 1H, H-23), 8.49 (s, 1H, H-12), 8.26 (d, 1H, *J* = 6.9, H-6), 7.37 (d, 1H, *J* = 2.9 Hz, H-3), 7.14 (dd, 1H, *J* = 7.0, 3.0, H-4), 6.80 (d, 1H, *J* = 2.0 Hz, H-17), 6.62 (d, 1H, *J* = 2.0 Hz, H-19), 5.97–5.87 (m, 1H, H-21), 5.57 (s, 2H, H-13), 5.09–5.00 (m, 2H, H-22), 3.81 (s, 3H, H-24), 3.79 (s, 3H, H-25), 3.26 (d, 2H, *J* = 6.7 Hz, H-20); ^13^C NMR (100 MHz, DMSO-*d*_6_) *δ*_C_ 161.62 (C-7), 158.22 (C-5), 154.86 (C-16), 148.03 (C-10), 143.28 (C-15), 143.06 (C-2), 138.33 (C-21), 132.56 (C-11), 131.02 (C-14), 127.50 (C-3), 122.36 (C-12), 122.27 (C-18), 122.08 (C-19), 121.66 (C-4), 118.12 (C-1), 116.06 (C-22), 113.98 (C-4), 112.88 (C-17), 105.92 (C-6), 56.34 (C-24), 55.96 (C-25), 49.41 (C-13), 39.49 (C-20). ESI-HRMS (+) m/z: Anal. Cal. for (C_22_H_21_N_5_0_4_) (M + H)^+^: 420.1672: found: 420.1669.

2-(1-(5-allyl-2-hydroxy-3-methoxybenzyl)-1*H*-1,2,3-triazol-4-yl)-6-fluoroquinazolin-4(3*H*)-one (**10c**): Light gray solid. Quantitative yield. ^1^H NMR (400 MHz, DMSO-*d*_6_) *δ*_H_ 1H 12.53 (s, 1H, H-8), 9.07 (s, 1H, H-23), 8.68 (s, 1H, H-12), 7.90–7.60 (m, 3H, H-3, H-4 and H-6), 6.83 (d, 1H, *J* = 2.1 Hz, H-17), 6.70 (d, *J* = 2.1 Hz, 1H, H-19), 5.94 (dd, *J* = 23.8, 10.0 Hz, 1H, H-21), 5.56 (s, 2H, H-13), 5.08–5.00 (m, 2H, H-21), 3.80 (s, 3H, H-24), 3.28 (s, 2H, H-20); ^13^C NMR (100 MHz, DMSO-*d*_6_) *δ*_C_ 160.45 (d, *J* = 244 Hz, C-5), 160.85 (C-7), 148.07 (C-16), 143.26 (C-10), 141.44 (C-15), 138.30 (C-21), 131.06 (C-14), 126.59 (C-19), 123.47 (d, *J* = 24 Hz, C-4), 122.02 (C-12), 121.80 (C-1), 116.12 (C-4), 113.00 (C-3), 111.29 (C-17), 111.18 (d, *J* = 23 Hz, C-6), 56.33 (C-24), 49.52 (C-13), 39.03 (C-20). ESI-HRMS (+) m/z: Anal. Cal. for (C_21_H_18_FN_5_O_3_) (M + H)^+^: 408.1472: found: 408.1469.

### 3.2. Biological Tests

#### 3.2.1. Antifungal and Antibacterial Assays

##### Compound Preparation

All tested compounds (**9a**–**9d**, **10a**–**10c**, **13**, **14**, and eugenol (**1**)) were prepared in DMSO (Sigma-Aldrich, St. Louis, MO, USA) at a concentration of 10 mg/mL. The reference drugs used (voriconazole (Sigma-Aldrich, St. Louis, MO, USA) for fungi and quality control; gentamicin (Sigma-Aldrich, St. Louis, MO, USA) for bacterial and quality control) were prepared at 6.4 mg/mL. The stock solutions of the compounds were stored at –20 °C until immediately before the assays and then were diluted in fresh culture medium, RPMI-1640 medium (Biochrom AG, Berlin, Germany) buffered with 3-(*N*-morpholino)propanesulfonic acid (MOPS—Sigma-Aldrich, St. Louis, MO, USA) for fungi and cation-adjusted Mueller–Hinton broth (MHBII—Becton Dickinson, France) for bacteria, henceforth referred to as RPMI and MHB, respectively.

##### Fungal and Bacterial Strains

Fungal strains, including reference strains and clinical isolates, were used for the study of the antifungal activity: a yeast reference strain from American Type Culture Collection (ATCC), *Candida albicans* ATCC 10231; filamentous fungi reference strain *Aspergillus fumigatus* ATCC 240305; and a clinical isolate of dermatophytes *Trichophyton rubrum* FF5. *Candida krusei* ATCC 6258 was used as a quality control. All fungal strains were stored in Sabouraud dextrose broth (SDB—Bio-Mèrieux, Marcy L’Etoile, France) with glycerol (20%) at −80 °C and were subcultured in Sabouraud dextrose agar (SDA—Bio-Mèrieux, Marcy L’Etoile, France) for 24–72 h (yeasts and *A. fumigatus*) or 5–7 days (*T. rubrum*) before each assay to obtain optimal growth and purity conditions. Two bacteria strains were used, one Gram positive and one Gram negative: *Staphylococcus aureus* ATCC 25923 and *Escherichia coli* ATCC 25922, respectively. The bacterial strains were stored in trypticase soy broth (TSB—Biolife, Milan, Italy) with glycerol (10%) at −80 °C and subcultured in Mueller–Hinton agar (MHA—Bio-Mèrieux, Marcy L’Etoile, France) for 24 h before each assay.

##### Antifungal Activity

For the purpose of quantitatively measuring the antifungal activity in vitro, a broth microdilution method was used to determine the MICs using the CLSI reference protocols M27-A3 [27] for yeasts and M38-A2 [28] for filamentous fungi. In short, two-fold serial dilutions of each stock solution were prepared using RPMI medium (pH 7) to obtain a range of test concentrations from 16 to 512 μg/mL. The yeasts or spore suspensions were prepared from 24 to 72-h cultures (yeasts and *Aspergillus*) or from 5–7 days of cultures (dermatophyte) in saline solution (with a drop of Tween 20 for the filamentous fungi). The transmittance of the cell density of yeasts was adjusted to 0.5 McFarland standard, and for filamentous fungi, the spores were counted using a Neubauer camera. Dilutions were performed using RPMI to obtain cell final concentrations in the plate of 0.5–2.5 × 10^3^ colony forming units (CFU)/mL for yeasts, 0.4–5 × 10^4^ CFU/mL for *Aspergillus,* and 1–3 × 10^3^ CFU/mL for dermatophytes. Plates with the prepared serial dilutions of the tested compounds were inoculated with the same volume of the fungal suspension, and sterility (wells with only culture medium), growth (wells with cell suspensions in culture medium), and DMSO controls (wells with cell suspensions in culture medium containing 1% DMSO) were included in each assay. Immediately thereafter, the plates were incubated in a humid atmosphere for 48 h at 36 °C for yeasts and *Aspergillus* or for 5–7 days at 26 °C for dermatophytes, and the antifungal activity was determined by MIC values, read visually (according to the CLSI norms). For the tested compounds, MIC values were considered the minimum concentration that inhibited the yeast growth by 100% compared to the growth control. The reference drug, voriconazole, was tested against *C. krusei* ATCC 6258 as a quality control, and the results were within the limits of CLSI reference documents.

Additionally, the minimum lethal concentration (MLC) was tested for some of the compounds that showed inhibitory activity, with the objective of evaluating the fungicidal potential of new compounds. After MIC readings, 10 μL of suspension were collected from wells corresponding to the MIC and the highest concentration following the MIC and were deposited in SDA plates. The plates were incubated following the conditions previously mentioned for the MIC evaluation, and the MLC was determined as the lowest concentration at which no fungal growth is observed.

##### Antibacterial Activity

For bacteria, MICs were determined by the broth microdilution method, following the recommendations of the reference method M100-S25 [29].

Similarly, as previous described, two-fold serial dilutions of each stock solution were prepared using MHB to obtain a range of test concentrations from 16 to 512 μg/mL. Bacterial cell suspensions were prepared from 24-h cultures in saline solution, and the transmittance of cell density was adjusted to 0.5 McFarland standard. Dilutions were performed using MHB to obtain cell final concentrations in the plate of 0.5–2.5 × 10^4^ CFU/mL.

Plates with the prepared serial dilutions of the tested compounds were inoculated with the same volume of the bacterial suspension, and sterility, growth and DMSO controls were included in each assay. Immediately thereafter, the plates were incubated in a humid atmosphere (aerobic environment) for 18–24 h at 36 °C.

MIC values were considered the minimum concentrations that inhibited the bacterial growth by 100% compared to the growth control. The reference drug, gentamicin, was tested as a quality control, and the results were according to the norms.

MLC was evaluated for some of the tested compounds that showed inhibition. A volume of 10 μL of suspension was collected from wells corresponding to the MIC and the highest following concentration and was deposited in MHA plates. The plates were incubated for 24 h at 36 °C, and the MLC was determined as the lowest concentration at which no bacterial growth was observed.

#### 3.2.2. Antiparasitic Assays

##### Parasite Cultures

Promastigotes from the *L. infantum* strain (MHOM/MA/67/ITMAP-263) were grown in 5-mL T25 flasks in Schneider’s insect medium supplemented with 10% heat-inactivated fetal bovine serum (FBS), 200 U/mL penicillin/streptomycin, 6 µg/mL Phenol Red, and 5 mM HEPES. The cultures were maintained in an incubator at 27 °C and diluted to 2 × 10^5^/mL every 5 days. For the assays, the parasites used were equivalent to late/log with 2 or 3 days of culture.

*T. brucei* Lister 427 bloodstream forms were grown in a humidified incubator at 37 °C and 5% CO_2_ in complete HMI-9 medium [30] supplemented with 10% heat-inactivated fetal bovine serum (FBS) and 100 UI/mL penicillin/streptomycin. Parasite maintenance was performed in T25 ventilated flasks by subpassage at a concentration of 1 × 10^4^/mL every 2 days in T25 ventilated flasks. Luciferase-expressing *L. infantum* (MHOM/MA/67/ITMAP-263) axenic amastigotes expressing episomal luciferase were maintained in MAA20 [31] at 37 °C in a 5% CO_2_ environment with subculture every 7 days at 1 × 10^6^/mL in 5-mL T25 ventilated flasks.

##### Anti-*T. brucei* Activity

The compounds’ efficacy against bloodstream-stage trypomastigotes was evaluated using a resazurin-based assay. Parasites were added to 100 µL of serial dilutions of compounds in supplemented complete medium at a cell density of 5 × 10^3^/mL. As a quality control, a dose–response curve for the antitrypanosomal pentamidine was included in all the assays. The final volume of the assay was 200 µL/well. Each condition was carried out in duplicate. Following 72 h of incubation at the specific conditions for parasites, 20 samples of a 0.5 mM resazurin solution was added, and the plates were incubated for a further 4 h under the same conditions. Fluorescence was measured at 544 nm and 590 nm excitation and emission wavelength, respectively, using a Synergy 2 Multi-Mode Reader (Biotek, Winooski, VT, USA). The results are shown as % of parasite growth inhibition compared to control (untreated parasites) and represent the average of at least three independent experiments. The effect was evaluated by the determination of the IC_50_ value (concentration required to inhibit growth in 50%) and calculated by non-linear regression curves using GraphPad Prism software, version 8.1.1 for Windows (GraphPad Software, San Diego, CA, USA). 

##### Anti-Leishmania Activity

The compounds’ efficacy against *L. infantum* promastigotes was evaluated using a resazurin-based assay. Parasites were added to 100 µL of serial dilutions of compounds in supplemented complete medium at a cell density of 5 × 10^5^/mL. As a quality control, a dose–response curve to the antileishmanial drug miltefosine was included in all the assays. The final volume of the assay was 200 µL/well. Each condition was carried out in duplicate. Following 72 h of incubation at the specific conditions for parasites, 20 µL of a 0.5 mM resazurin solution was added, and the plates were incubated for a further 4 h under the same conditions. Fluorescence was measured at 544 nm and 590 nm excitation and emission wavelengths, respectively, using a Synergy 2 Multi-Mode Reader (Biotek, Winooski, VT, USA). Results are shown as % of parasite growth inhibition compared to control (untreated parasites) and represent the average of at least three independent experiments. The effect was evaluated by the determination of the IC_50_ value (concentration required to inhibit growth in 50%) and was calculated by non-linear regression curves using GraphPad Prism software, version 8.1.1 for Windows (GraphPad Software, San Diego, CA, USA).

#### 3.2.3. Cytotoxicity Assay

A human leukemia cell line, THP-1 (ATCC^®^ TIB-202™), was cultured in RPMI-1640 medium supplemented with 10% heat-inactivated fetal bovine serum (FBS), 2 mM L-glutamine, 100 IU/mL penicillin/streptomycin, and 20 mM HEPES. The cell line was maintained in a humidified incubator at 37 °C and 5% CO_2_ by subculture every 3 days in 20 mL of media at a concentration of 2 × 10^5^/mL in a T75 flask. All cell culture reagents were purchased from Lonza-Bioscience (Morrisville, NC, USA).

The cytotoxicity effect of compounds on THP-1-derived macrophages was assessed by colorimetric MTT assay (3-(4,5-dimethylthiazol-2-yl)-2,5-diphenyl tetrazolium bromide). Briefly, THP-1 cells were suspended in RPMI complete medium at a density of 1 × 10^6^ cells/mL and 100 μL/well and were seeded in a 96-well plate and differentiated into macrophages by addition of 40 ng/mL of phorbol-myristate 13-acetate (PMA, Sigma, Saint Louis, MI, USA) for 24 h, followed by replacement with fresh medium for 24 more h. Subsequently, the cells were incubated with 100 μL of compounds ranging from 100 to 12.5 μM after dilution in the RPMI complete medium. Each condition was carried out in quadruplicate. After 72 h of incubation at 37 °C and 5% CO_2_, the medium was removed, and 200 μL of 0.5 mg/mL MTT solution diluted in RPMI was added. The plates were incubated for an additional 4 h. Then, 160 μL of media was removed, and the same volume of 2-propanol was added. Absorbance was read at 570 nm using a Synergy 2 Multi-Mode Reader (Biotek, Winooski, VT, USA). Cytotoxicity was evaluated by the determination of the CC_50_ value (drug concentration that reduced the percentage of viable cells by 50%) and calculated by non-linear regression analysis using GraphPad Prism software, version 8.1.1 for Windows (GraphPad Software, San Diego, CA, USA). The results represent the average of at least three independent experiments. For each compound, the selectivity index (SI) was calculated as the ratio between cytotoxicity in THP-1 (CC_50_, 72 h) and activity against parasites (IC_50_, 72 h).

## 4. Conclusions

New compounds, designed by molecular hybridization from a marine quinazolinone and eugenol, were synthesized and evaluated against protozoan species involved in neglected parasitic diseases and as possible antifungal and antibacterial agents. Initially we hypothesized that the marine quinazolinone described as having antifungal activity (**a**, Figure 1) could benefit from hybridization with eugenol. Moreover, the conjugation through a triazole moiety, critical for azole antifungal drugs, was hypothesized to increase the potential antifungal activity. In contrast to our expectations, no antifungal activity was detected for the series. Although no hybrid showed relevant antimicrobial action, inspired in our previous studies with indolylmethylpyrazinoquinazolines (**b**, Figure 1), six of the substances tested (intermediates **9b**, **9c**, and **9d** and final products **10b**, **10c**, and **14**) presented mild antitrypanosomal activity. One of them, the fluorinated intermediate **9c**, additionally showed a relevant leishmanicidal effect. Natural products, including marine specialized metabolites, are one potential source from which novel trypanocidal compounds have been disclosed. Most of these compounds have activity against multiple (micro)organisms, which could limit their application. The breakthrough of this work was the discovery of derivatives with a simple quinazolinone scaffold selective against parasites without antibacterial and antifungal activities, which are synthetically accessible and without chiral centers, in contrast to previously reported antiparasitic alkaloids, such as the indolylmethylpyrazinoquinazolines. The structural pattern explored constitutes a relevant starting point for future optimization in an attempt to find marine-inspired candidates for leishmanicidal and trypanosomicidal drugs. 

## Figures and Tables

**Table 1:** Antiparasitic potential of **9a**, **9b**, **9c**, **9d**, **10a**, **10b**, **10c**, **13**, **14**, eugenol (**1**), pentamidine, and miltefosine. The antiparasitic activity was evaluated by the IC_50_ against *L. infantum* promastigotes and *T. brucei* bloodstream parasites, and the cytotoxicity was evaluated by the CC_50_ in the THP-1 cell line. The selectivity index (SI) for each parasite is also presented. The SI for the compounds was calculated by the ratio of the antiparasitic activity to the CC_50_ in THP-1.

Compounds	Anti-Parasitic Activity IC_50_ (µM) 95% CI		Cytotoxicity CC_50_ (µM) 95% CI	SI	
	*L. infantum* Promastigotes	*T. brucei*	THP-1	*L. infantum*	*T. brucei*
**9a**	>40	>40	>100	-	--
	-	-	-		
**9b**	>40	16.39	>100	-	>6
	-	(14.04–19.06)	-		
**9c**	7.54 (5.85–9.58)	21.03	45.82	6	2
		(15.93–28.62)	(38.04–55.30)		
**9d**	>40	15.92	> 100	-	>6
	-	(13.42–18.89)	-		
**10a**	>40	>40	>100	-	--
	-	-	-		
**10b**	>40	19.9	>100	-	>5
	-	(17.73–22.29)	-		
**10c**	>40	11.17	>100	-	>9
	-	(7.97–15.28)	-		
**13**	>40	>40	>100	-	--
	-	-	-		
**14**	>40	31.68	>100	-	>3
	-	(27.98–36.34)	-		
Eugenol	>40	>40	>100	-	-
	-	-	-		
Pentamidine	NT	0.0056	37.71 (31.75–43.57)		6734
		(0.0052–0.006)			
Miltefosine	10.98 (10.17–12.15)	NT	29.38 (23.97–36.02)	3	

NT: not tested; -: not determined.

## Data Availability

The data presented in this study are available in the article.

## Supplementary Materials

The following supporting information can be downloaded at: https://www.mdpi.com/article/10.3390/md21110551/s1, Figures S1–S42: ^1^H, ^13^C NMR and ESI-HRMS spectra of compounds **4**, **6a**, **7a**, **9a-9d**, **10a-10c**, **13** and **14** and antimicrobial activity of compounds **9a**–**9d**, **10a**–**10c**, **13**, **14**, and **eugenol**. Table S1: Antimicrobial activity of compounds **9a–9d, 10a–10c, 13, 14,** and **eugenol**.
