# Supplementary material for: Synthesis and Evaluation of Marine-Inspired Compounds Result in Hybrids with Antitrypanosomal and Antileishmanial Activities
Source: Mar Drugs. 2023 Oct 24;21(11):551. doi: 10.3390/md21110551 (PMC10671849; doi:10.3390/md21110551)
Supplement: Supplementary file 1 [file marinedrugs-21-00551-s001.zip › marinedrugs-2673400-supplementary.pdf]

Supplementary Material

Synthesis and evaluation of marine-inspired compounds result in hybrids with antitrypanosomal and antileishmanial activities

Diogo Teixeira Carvalho[1,2*], Melissa Teixeira[1,3], Sara Luelmo[4], Nuno Santarém[3,4], Eugénia Pinto[4], Anabela Cordeiro-da-Silva[3,4], Emília Sousa[1,5*]

[1]Laboratory of Organic and Pharmaceutical Chemistry, Department of Chemical Sciences, Faculty of Pharmacy, University of Porto, Rua Jorge Viterbo Ferreira 228, 4050-313 Porto, Portugal.
[2]Laboratory of Research in Pharmaceutical Chemistry, Department of Food and Drugs, Faculty of Pharmaceutical Sciences, Federal University of Alfenas, Rua Gabriel Monteiro da Silva, 700, 37130-001 Alfenas, Brazil.
[3]Laboratory of Microbiology, Department of Biological Sciences, Faculty of Pharmacy, University of Porto, Rua de Jorge Viterbo Ferreira 228, 4050-313 Porto, Portugal.
[4]Institute for Research and Innovation in Health (i3S), University of Porto, 4200-135 Porto, Portugal.
[5]Interdisciplinary Centre of Marine and Environmental Research (CIIMAR), Terminal de Cruzeiros do Porto de Leixões, Av. General Norton de Matos s/n, 4450-208 Matosinhos, Portugal.

* Correspondence: diogo.carvalho@unifal-mg.edu.br (D.C.); esousa@ff.up.pt (E.S.)

TABLE OF CONTENTES

**Figure S1.**	^{1}H NMR Spectrum for Compound **4** in $CDCl_3$ (300 MHz)	3
**Figure S2.**	^{13}C NMR Spectrum for Compound **4** in $CDCl_3$ (75 MHz)	3
**Figure S3.**	^{1}H NMR Spectrum for Compound **6a** in DMSO-d_6 (400 MHz)	4
**Figure S4.**	^{13}C NMR Spectrum for Compound **6a** in DMSO- d_6 (100 MHz)	4
**Figure S5.**	^{1}H NMR Spectrum for Compound **7a** in DMSO-d_6 (400 MHz)	5
**Figure S6.**	^{13}C NMR Spectrum for Compound **7a** in DMSO- d_6 (100 MHz)	5
**Figure S7.**	^{1}H NMR Spectrum for Compound **9a** in DMSO-d_6 (400 MHz)	6
**Figure S8.**	^{13}C NMR Spectrum for Compound **9a** in DMSO- d_6 (100 MHz)	6
**Figure S9.**	DEPT-135 Subspectrum for Compound **9a** in DMSO- d_6 (100 MHz)	7
**Figure S10.**	ESI-HRMS Spectrum for Compound **9a**	7
**Figure S11.**	^{1}H NMR Spectrum for Compound **9b** in DMSO-d_6 (400 MHz)	8
**Figure S12.**	^{13}C NMR Spectrum for Compound **9b** in DMSO- d_6 (100 MHz)	8
**Figure S13.**	DEPT-135 Subspectrum for Compound **9b** in DMSO- d_6 (100 MHz)	9
**Figure S14.**	ESI-HRMS Spectrum for Compound **9b**	9
**Figure S15.**	^{1}H NMR Spectrum for Compound **9c** in DMSO-d_6 (400 MHz)	10
**Figure S16.**	^{13}C NMR Spectrum for Compound **9c** in DMSO- d_6 (100 MHz)	10
**Figure S17.**	DEPT-135 Subspectrum for Compound **9c** in DMSO- d_6 (100 MHz)	11
**Figure S18.**	ESI-HRMS Spectrum for Compound **9c**	11
**Figure S19.**	^{1}H NMR Spectrum for Compound **9d** in DMSO-d_6 (400 MHz)	12
**Figure S20.**	^{13}C NMR Spectrum for Compound **9d** in DMSO- d_6 (100 MHz)	12
**Figure S21.**	DEPT-135 Subspectrum for Compound **9d** in DMSO- d_6 (100 MHz)	13
**Figure S22.**	ESI-HRMS Spectrum for Compound **9d**	13
**Figure S23.**	^{1}H NMR Spectrum for Compound **10a** in DMSO-d_6 (400 MHz)	14
**Figure S24.**	^{13}C NMR Spectrum for Compound **10a** in DMSO- d_6 (100 MHz)	14
**Figure S25.**	DEPT-135 Subspectrum for Compound **10a** in DMSO- d_6 (100 MHz)	15
**Figure S26.**	ESI-HRMS Spectrum for Compound **10a**	15
**Figure S27.**	^{1}H NMR Spectrum for Compound **10b** in DMSO-d_6 (400 MHz)	16
**Figure S28.**	^{13}C NMR Spectrum for Compound **10b** in DMSO- d_6 (100 MHz)	16
**Figure S29.**	DEPT-135 Subspectrum for Compound **10b** in DMSO- d_6 (100 MHz)	17
**Figure S30.**	ESI-HRMS Spectrum for Compound **10b**	17
**Figure S31.**	^{1}H NMR Spectrum for Compound **10c** in DMSO-d_6 (400 MHz)	18
**Figure S32.**	^{13}C NMR Spectrum for Compound **10c** in DMSO- d_6 (100 MHz)	18
**Figure S33.**	DEPT-135 Subspectrum for Compound **10c** in DMSO- d_6 (100 MHz)	19
**Figure S34.**	ESI-HRMS Spectrum for Compound **10c**	19
**Figure S35.**	^{1}H NMR Spectrum for Compound **13** in DMSO-d_6 (400 MHz)	20

**Figure S36.**	^{13}C NMR Spectrum for Compound **13** in DMSO- d_6 (100 MHz)	20
**Figure S37.**	DEPT-135 Subspectrum for Compound **13** in DMSO- d_6 (100 MHz)	21
**Figure S38.**	ESI-HRMS Spectrum for Compound **13**	21
**Figure S39.**	^{1}H NMR Spectrum for Compound **14** in DMSO-d_6 (400 MHz)	22
**Figure S40.**	^{13}C NMR Spectrum for Compound **14** in DMSO- d_6 (100 MHz)	22
**Figure S41.**	DEPT-135 Subspectrum for Compound **14** in DMSO- d_6 (100 MHz)	23
**Figure S42.**	ESI-HRMS Spectrum for Compound **14**	23
**Table S1.**	Antimicrobial activity of compounds **9a**-**9d**, **10a**-**10c**, **13**, **14**, and **eugenol**	24

Figure S1. ^{1}H NMR Spectrum for Compound **4** in $CDCl_3$ (300 MHz)

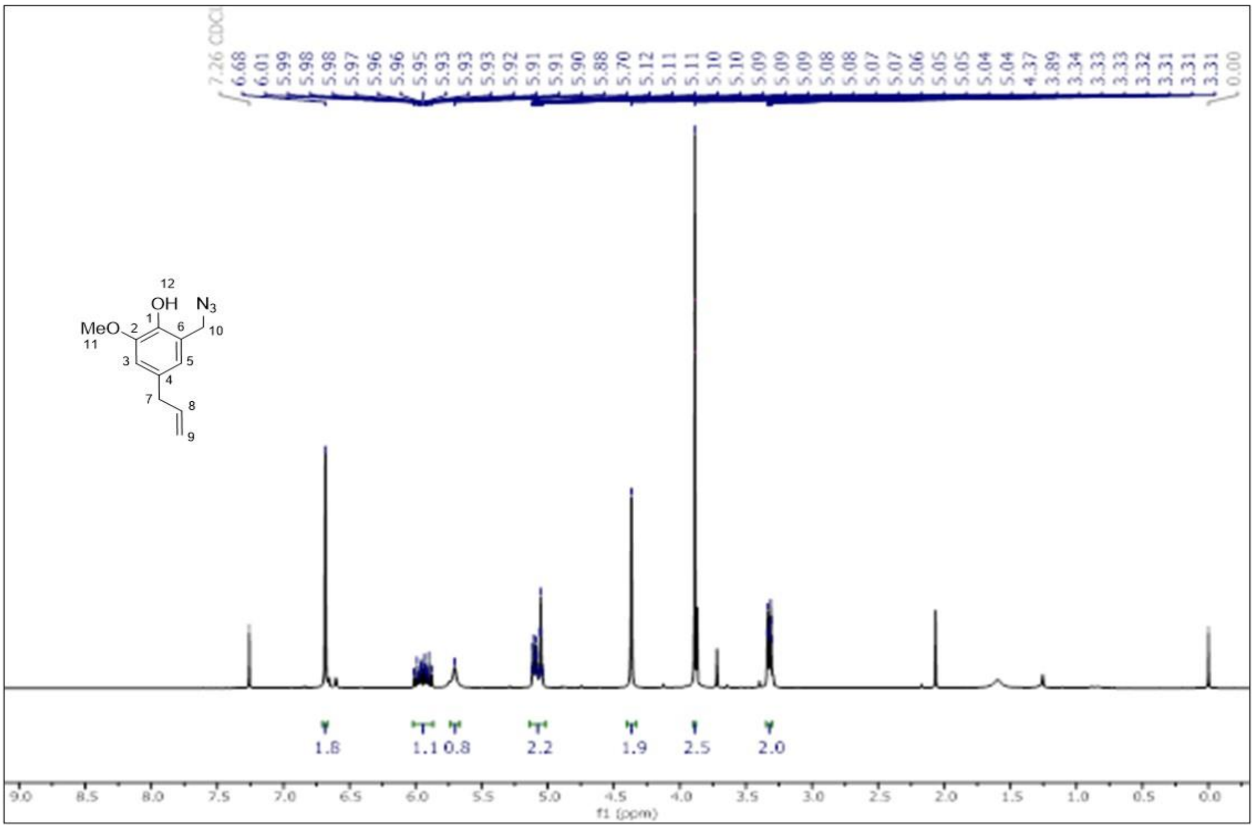

Figure S2. ^{13}C NMR Spectrum for Compound **4** in $CDCl_3$ (75 MHz)

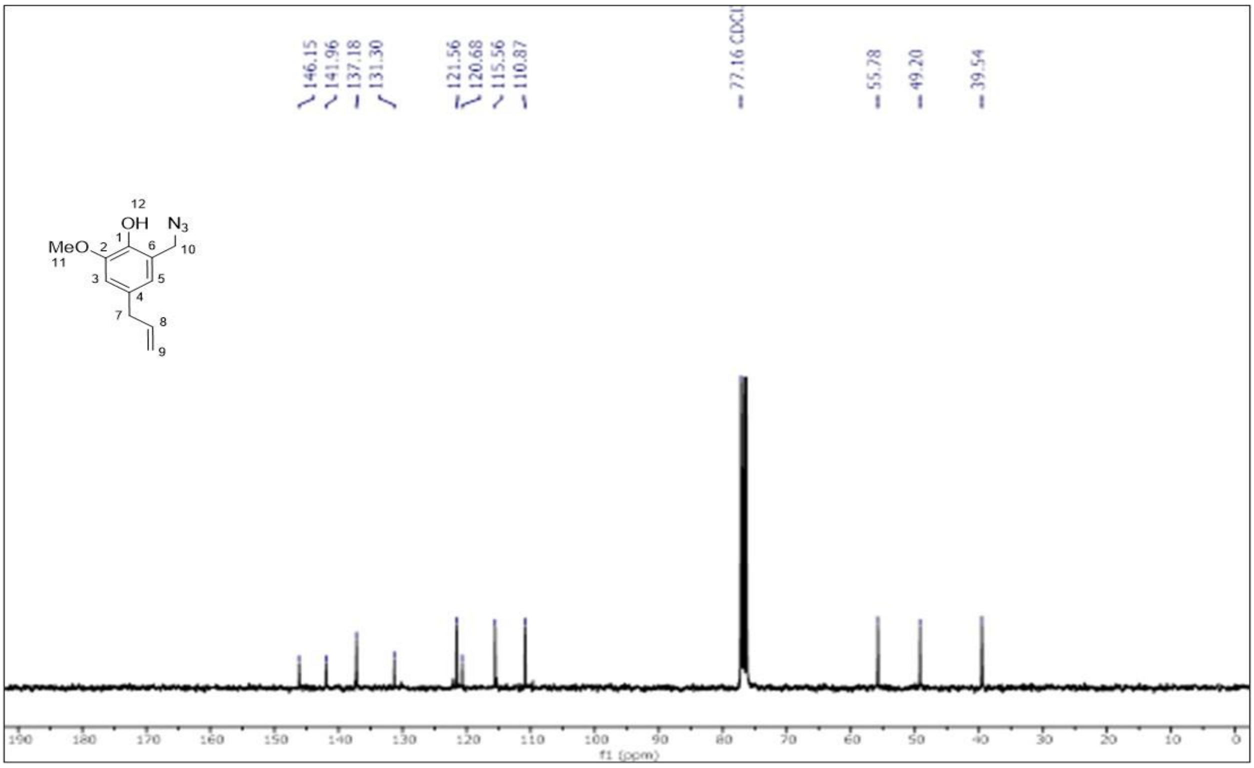

Figure S3. ^{1}H NMR Spectrum for Compound **6a** in DMSO-d_6 (400 MHz)

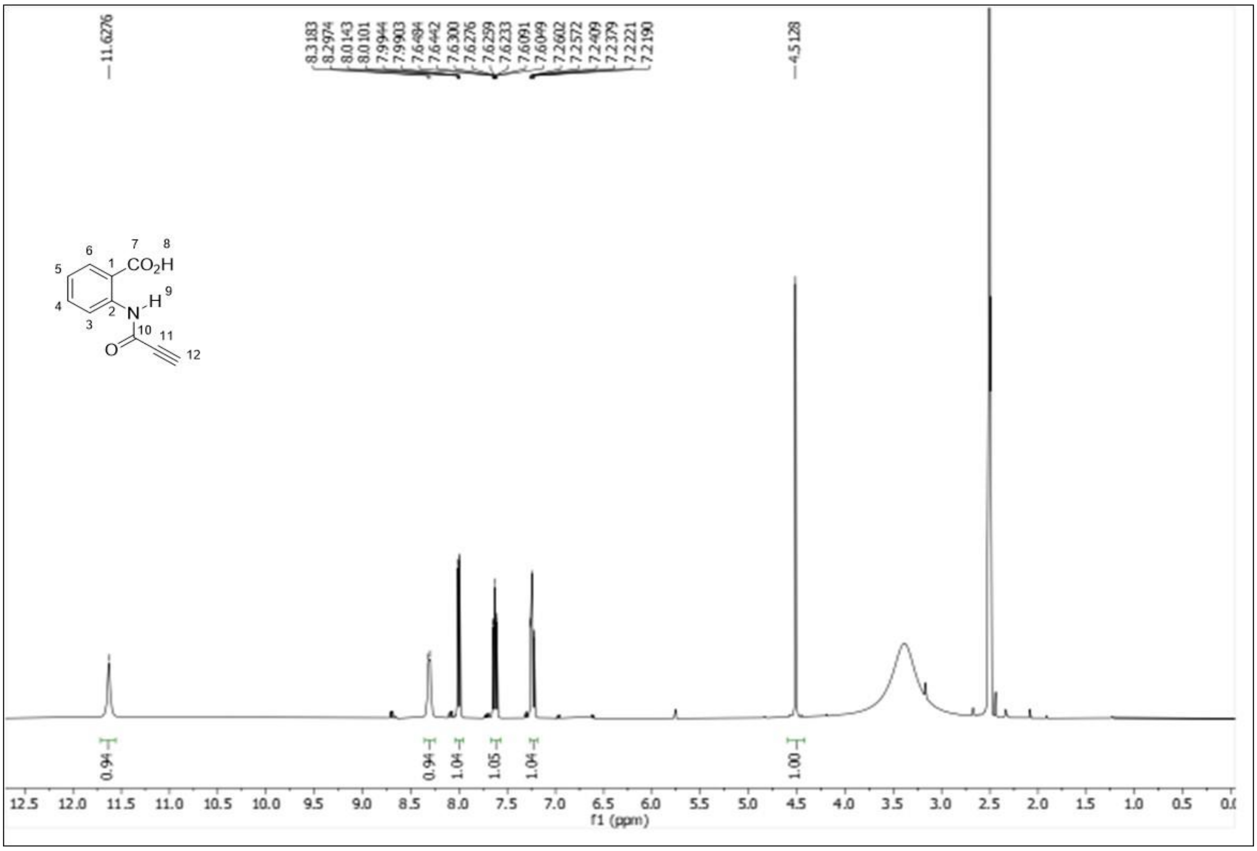

Figure S4. ^{13}C NMR Spectrum for Compound **6a** in DMSO-d_6 (100 MHz)

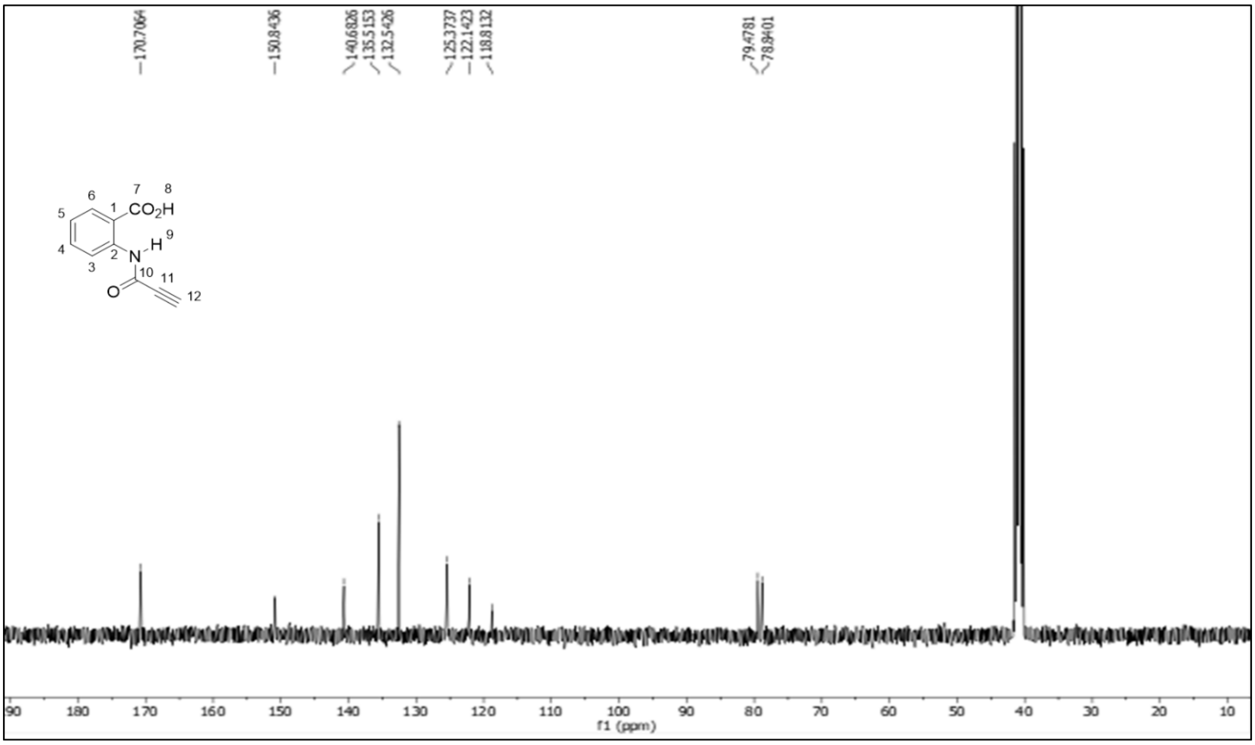

Figure S5. ^{1}H NMR Spectrum for Compound **7a** in DMSO-d_6 (400 MHz)

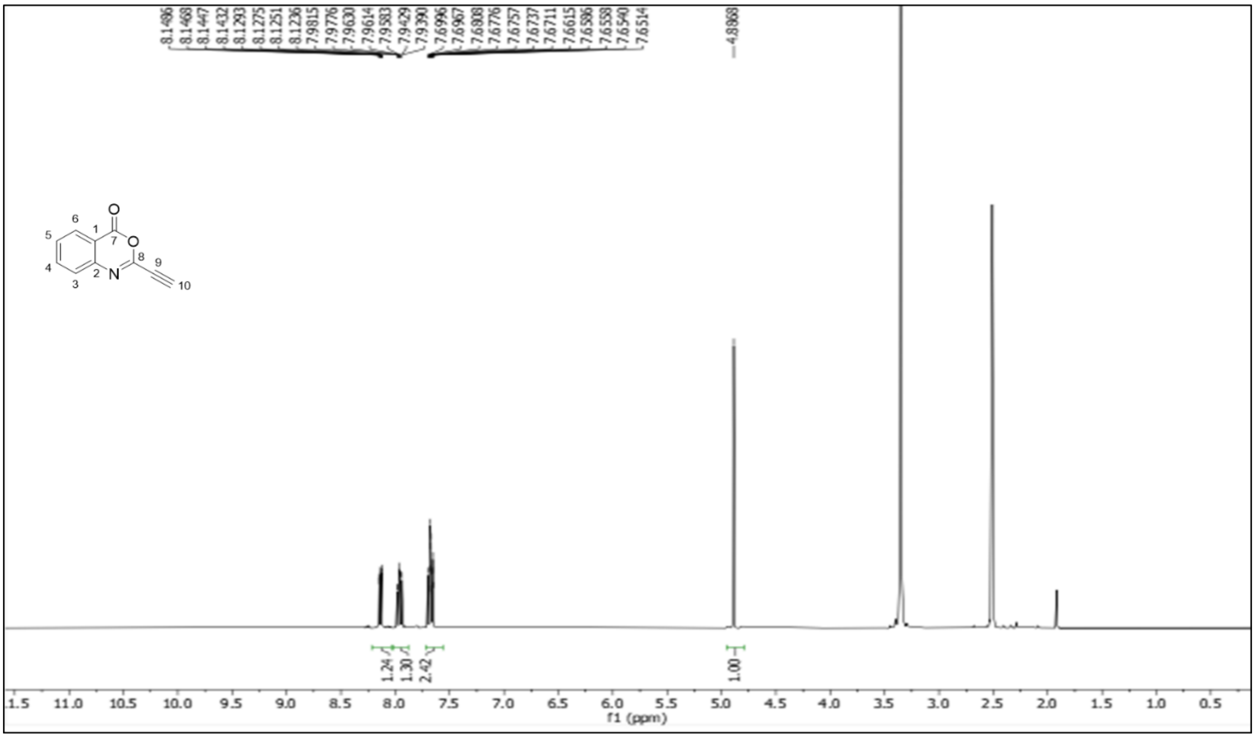

Figure S6. ^{13}C NMR Spectrum for Compound **7a** in DMSO-d_6 (100 MHz)

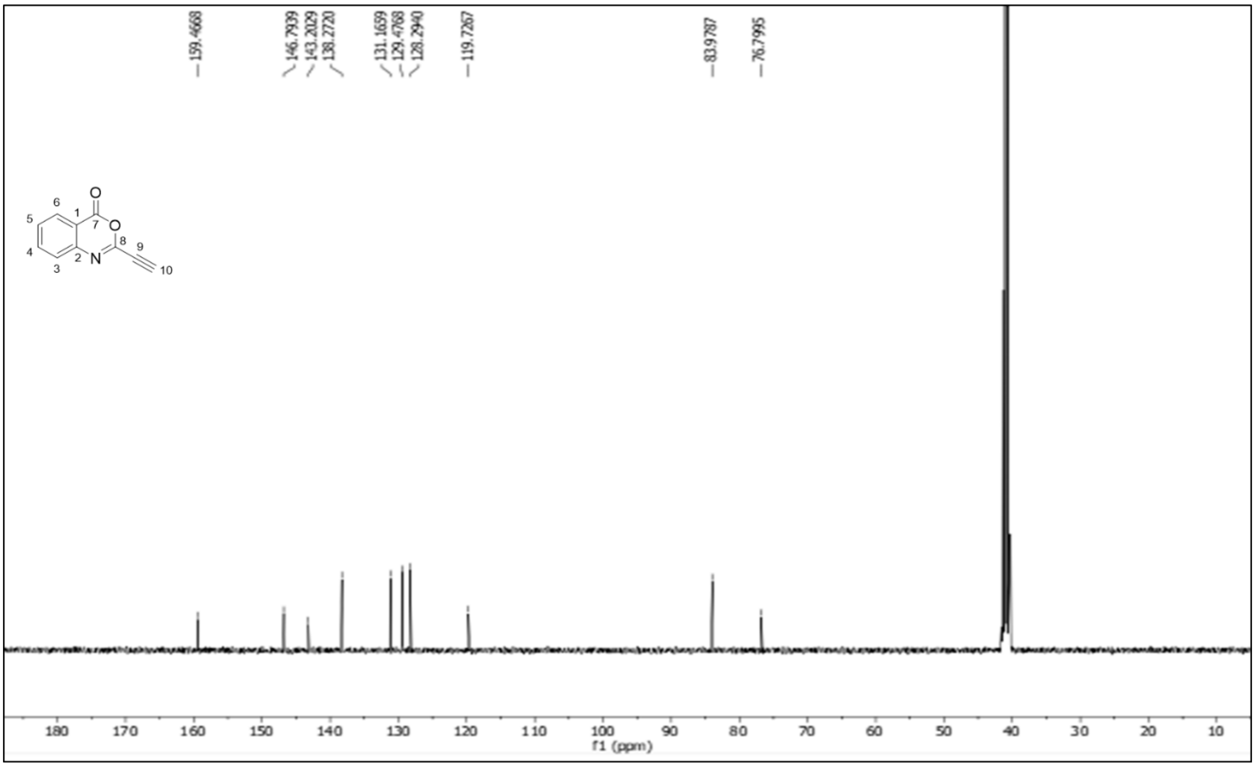

Figure S7. ^{1}H NMR Spectrum for Compound **9a** in DMSO-d_6 (400 MHz)

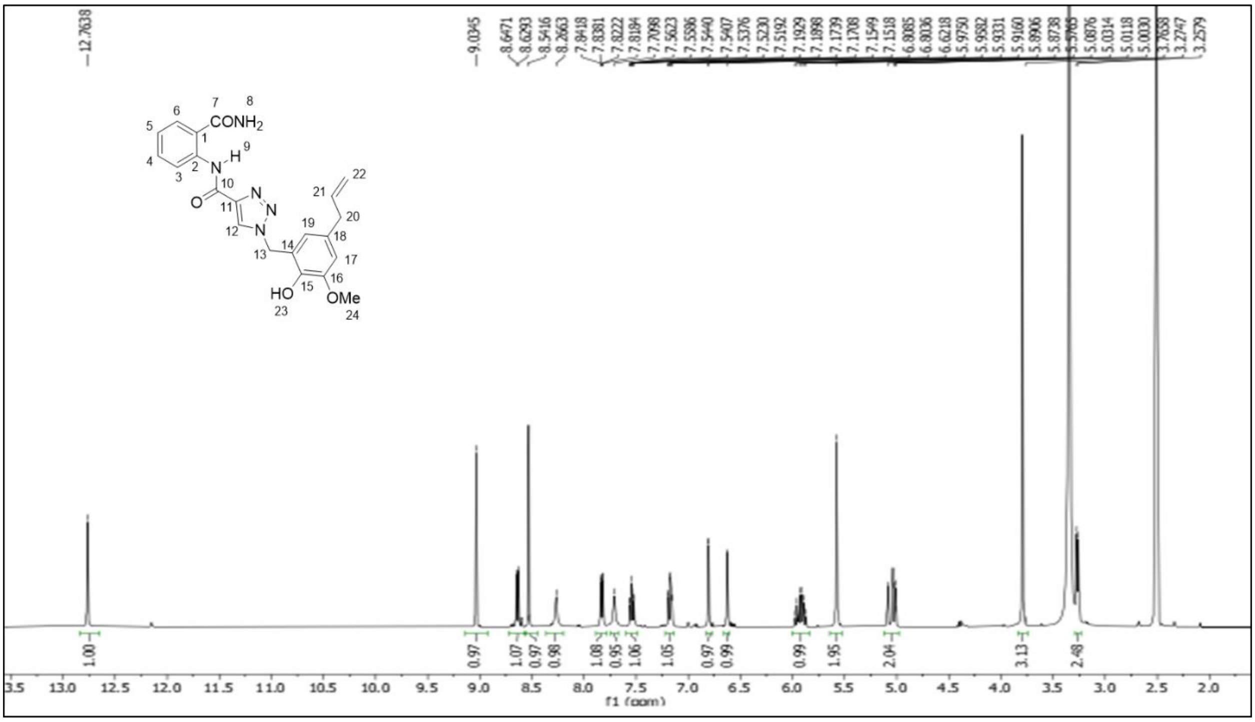

Figure S8. ^{13}C NMR Spectrum for Compound **9a** in DMSO-d_6 (100 MHz)

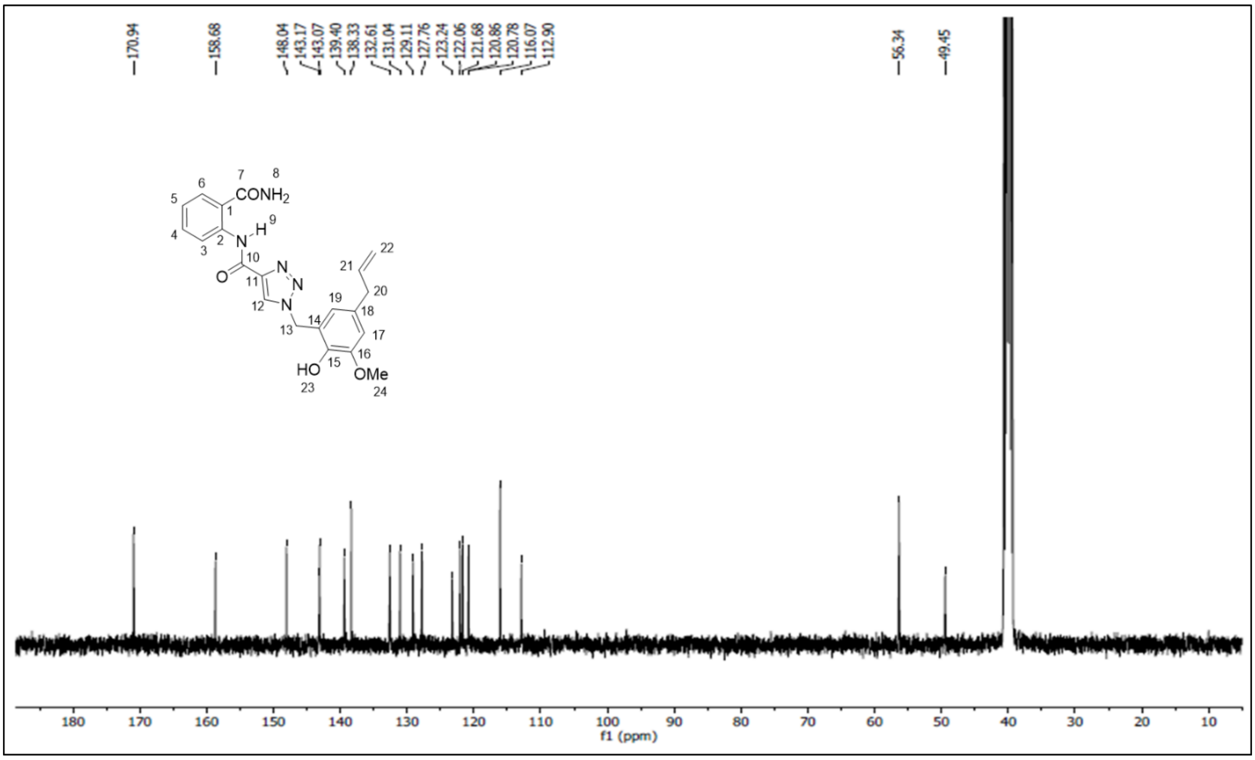

Figure S9. DEPT-135 Subspectrum for Compound **9a** in DMSO-d_6 (100 MHz)

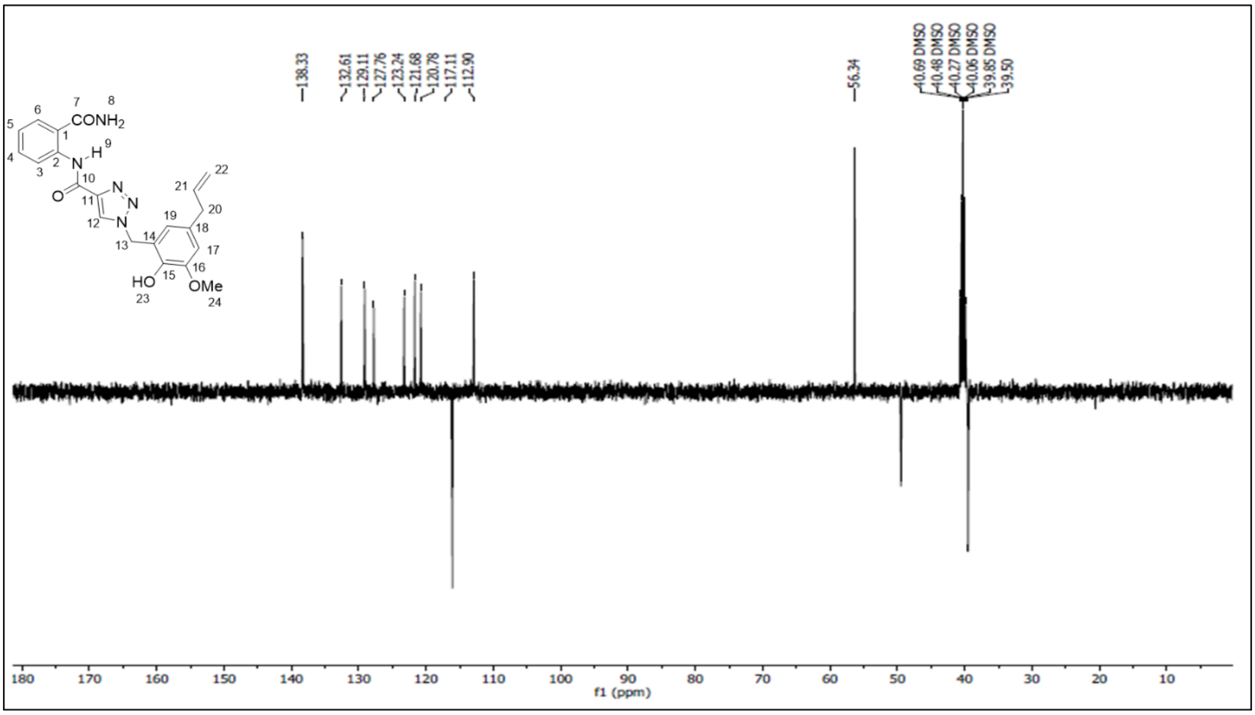

Figure S10. ESI-HRMS Spectrum for Compound **9a**

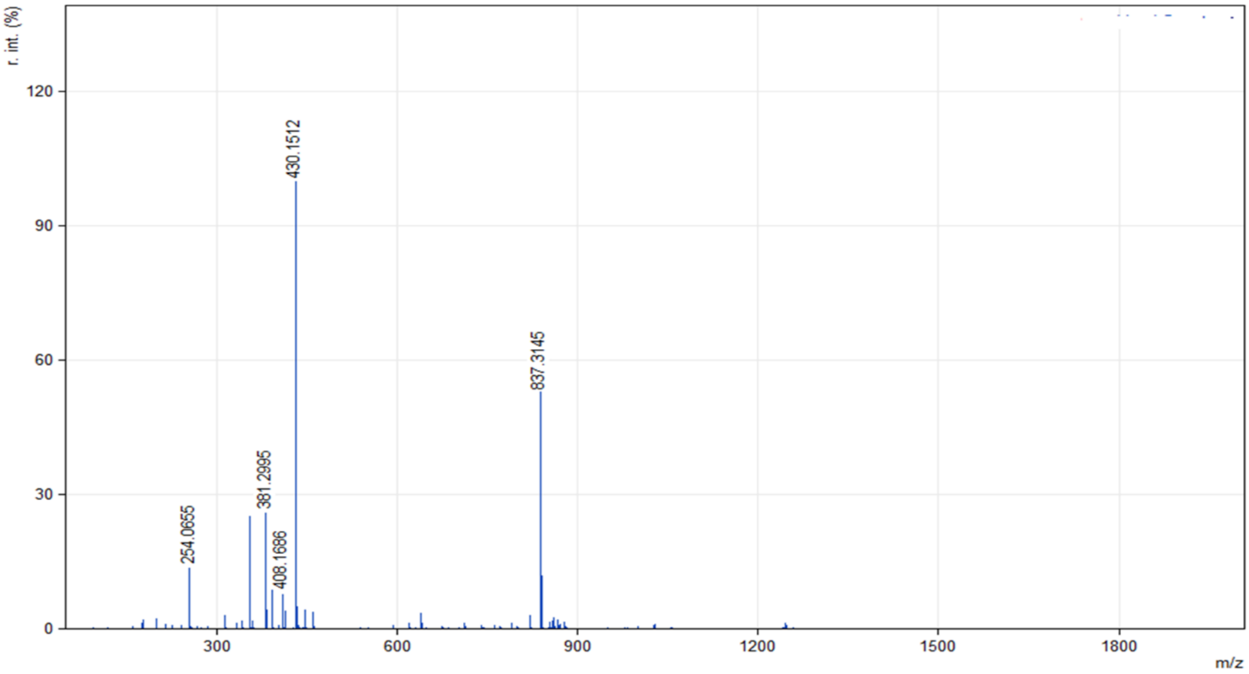

Figure S11. ^{1}H NMR Spectrum for Compound **9b** in DMSO-d_6 (400 MHz)

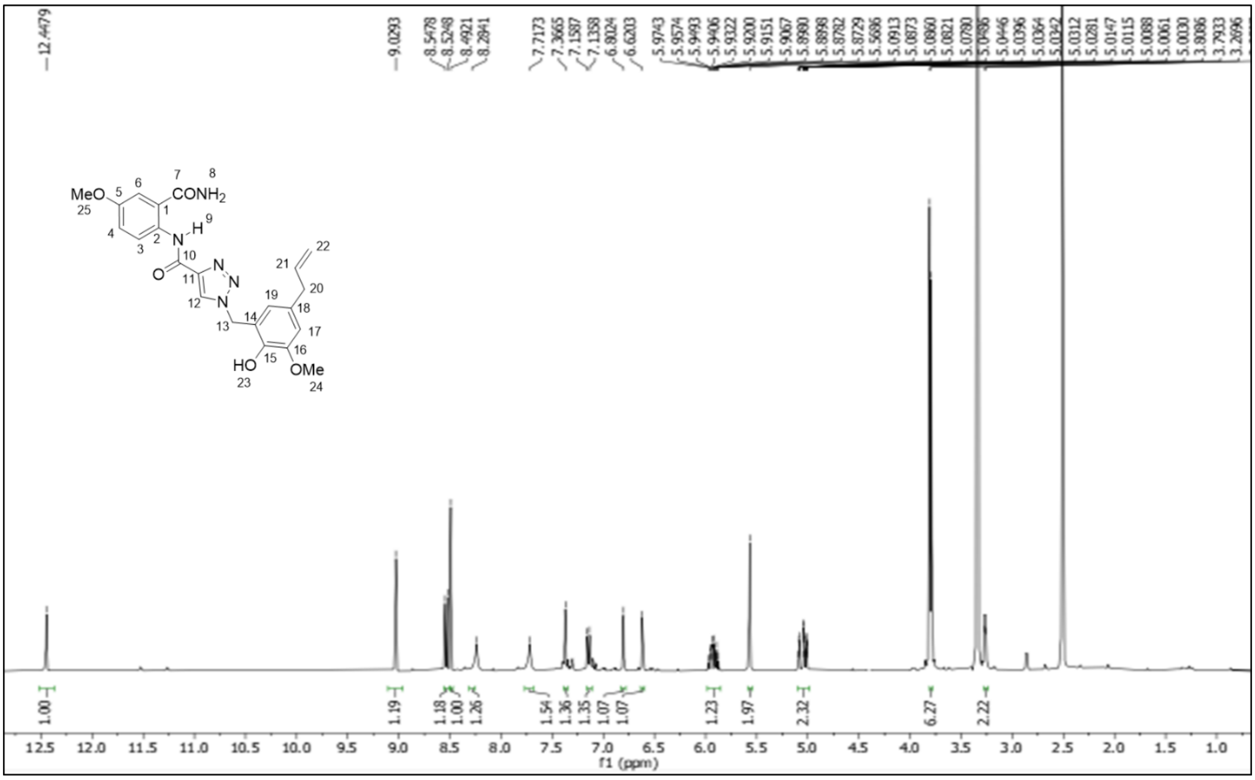

Figure S12. ^{13}C NMR Spectrum for Compound **9b** in DMSO-d_6 (100 MHz)

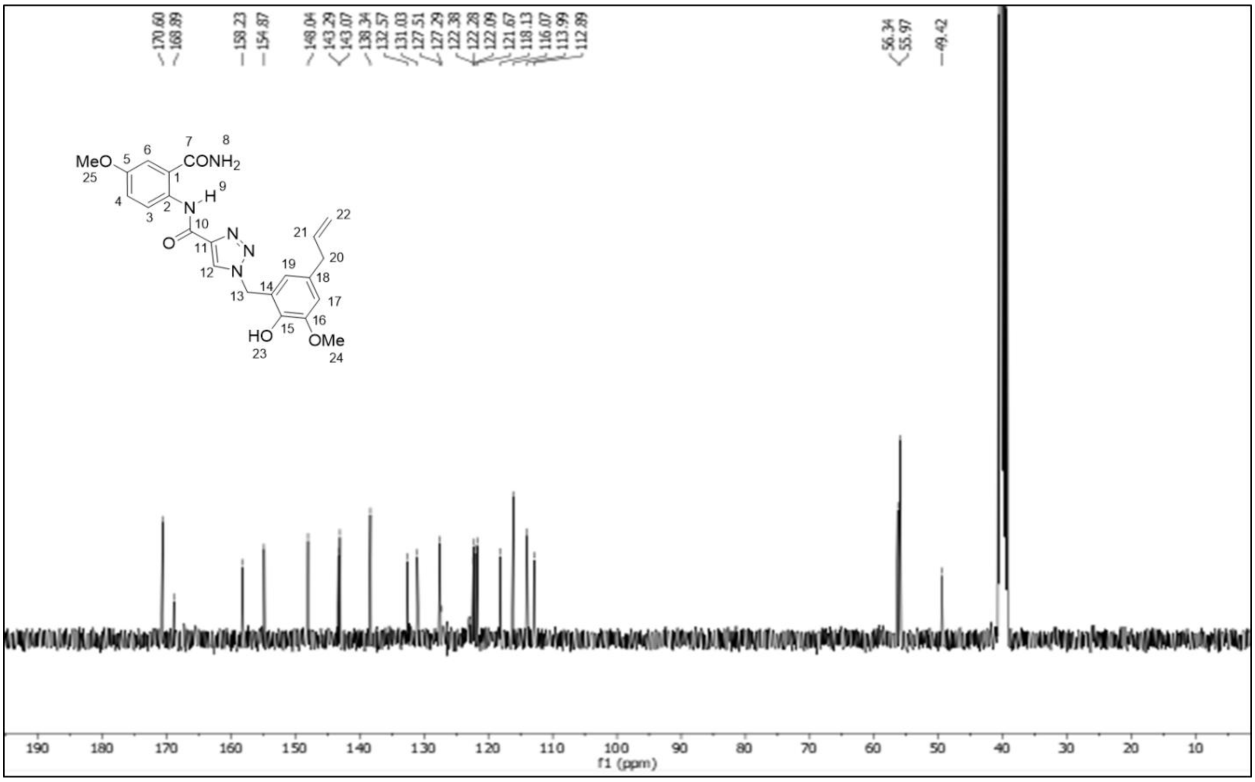

Figure S13. DEPT-135 Subspectrum for Compound **9b** in DMSO-d_6 (100 MHz)

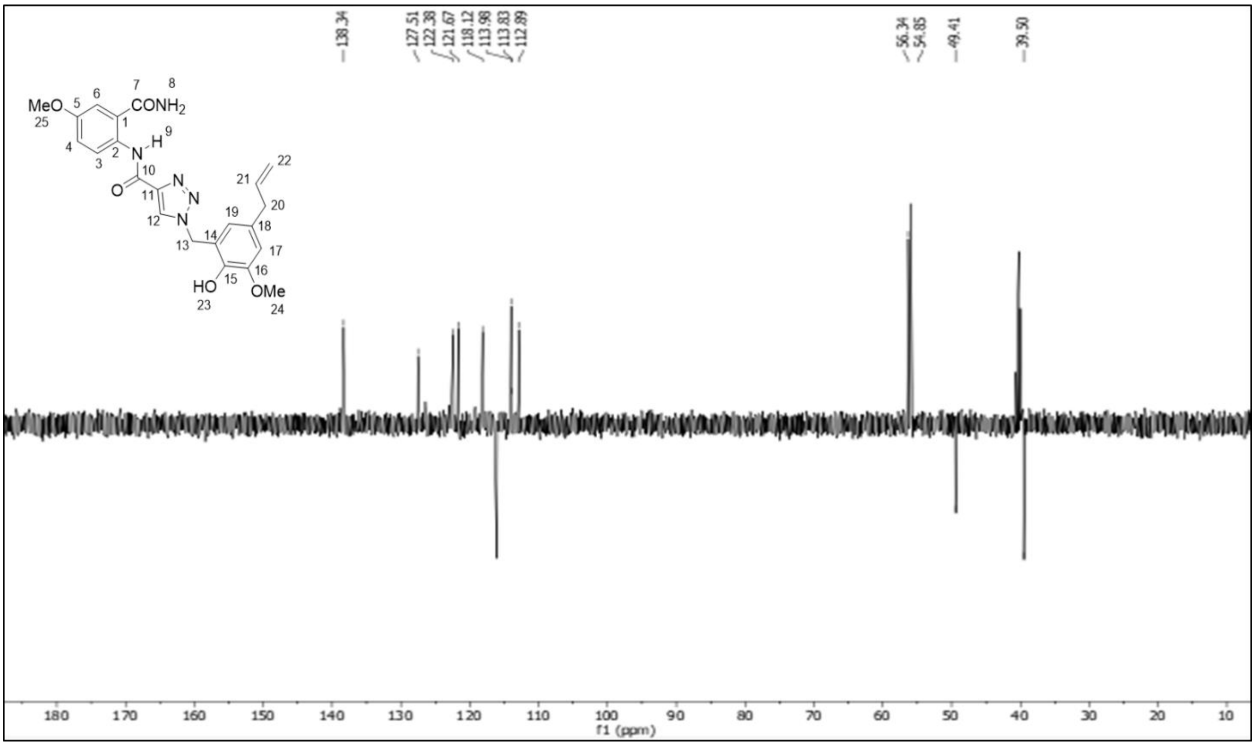

Figure S14. ESI-HRMS Spectrum for Compound **9b**

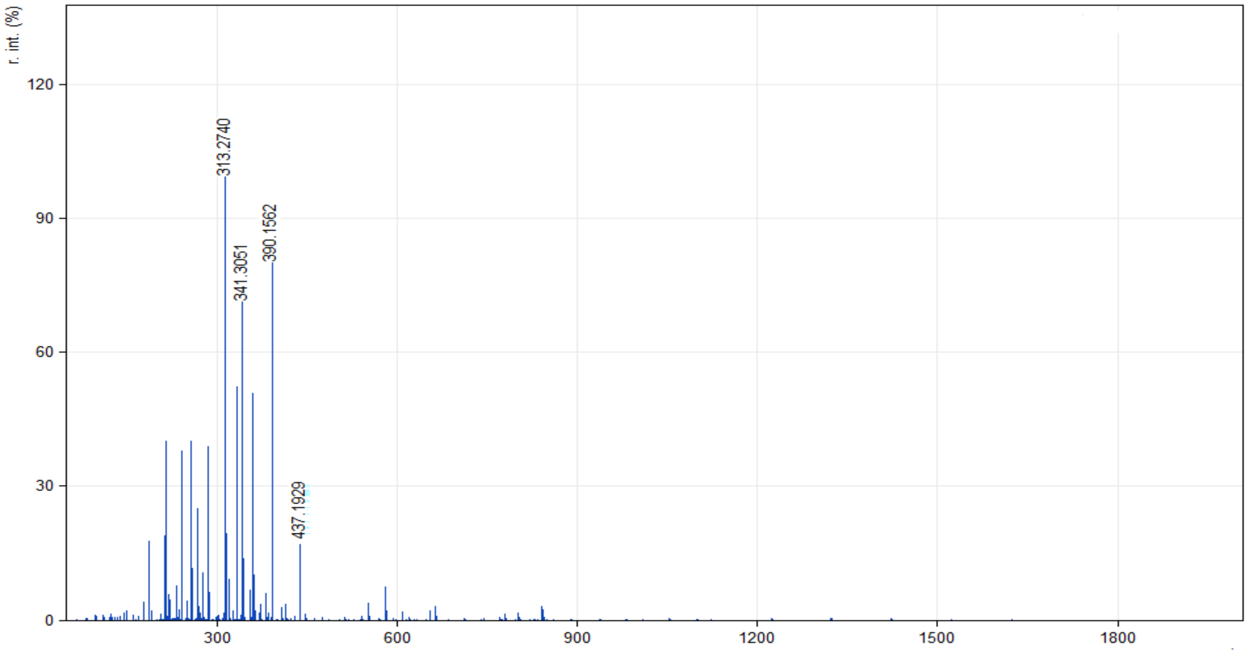

Figure S15. ^{1}H NMR Spectrum for Compound **9c** in DMSO-d_6 (400 MHz)

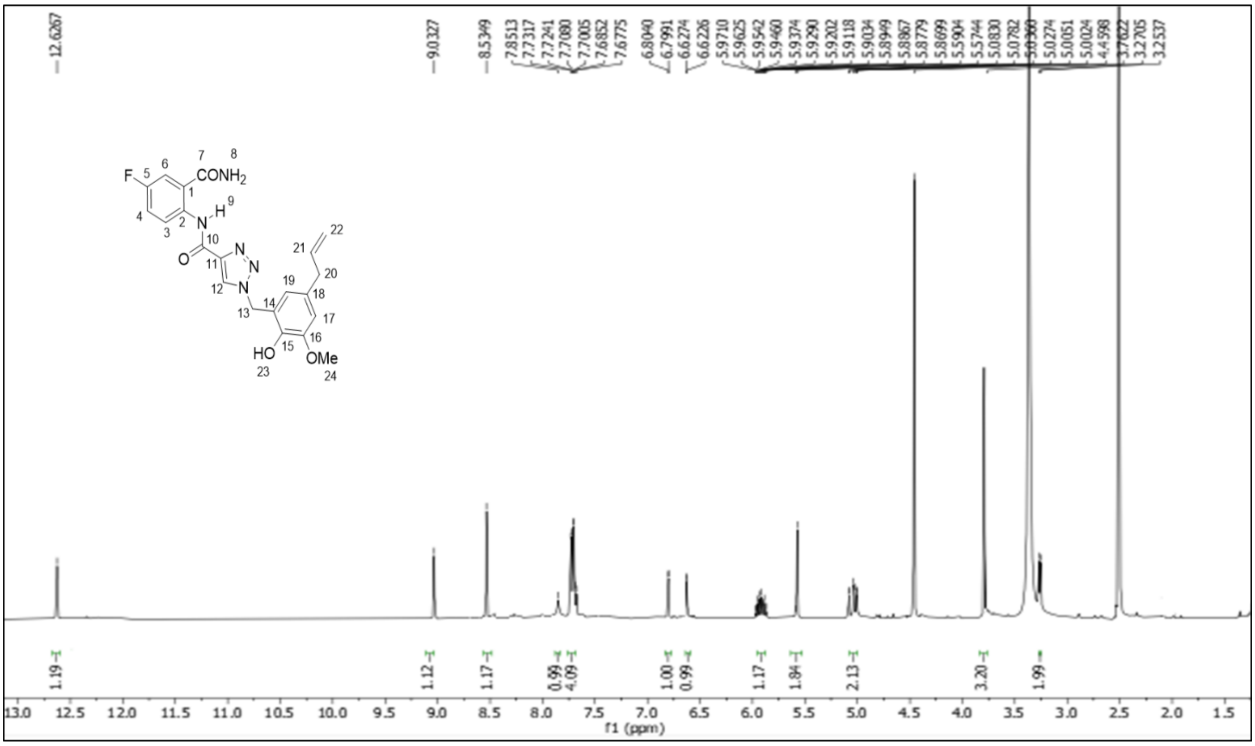

Figure S16. ^{13}C NMR Spectrum for Compound **9c** in DMSO-d_6 (100 MHz)

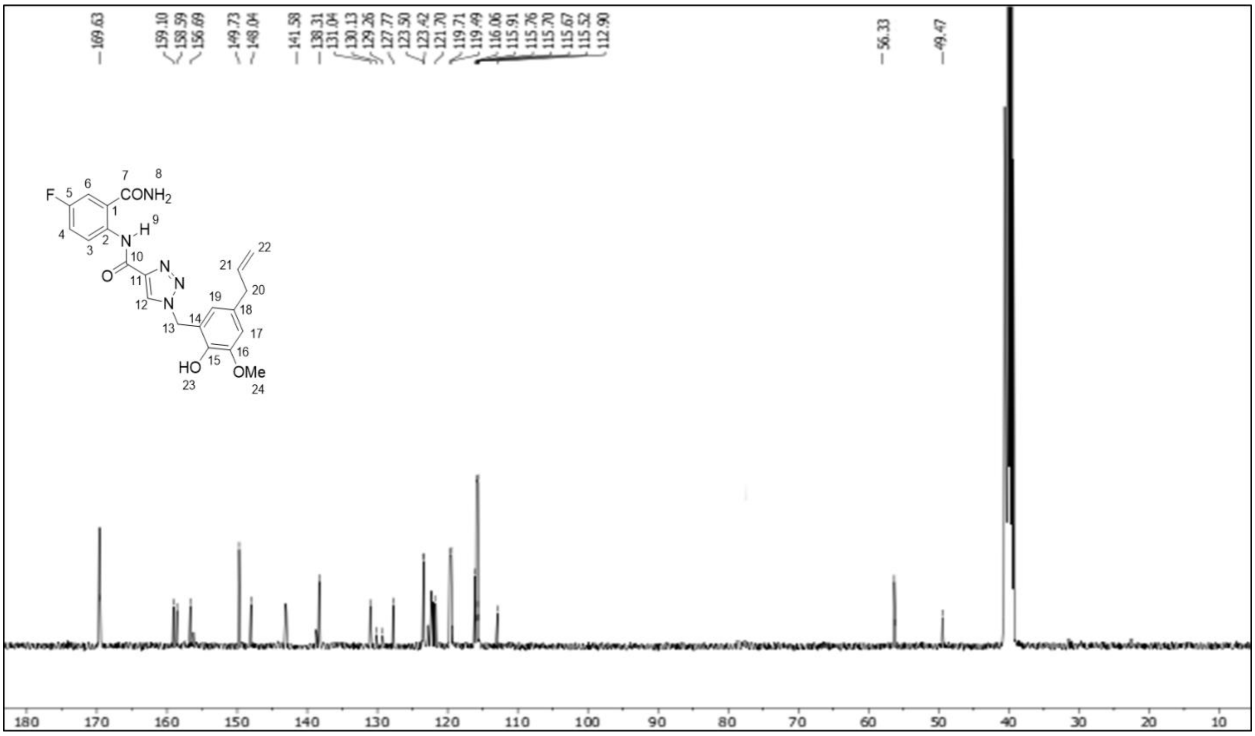

Figure S17. DEPT-135 Subspectrum for Compound **9c** in DMSO-d_6 (100 MHz)

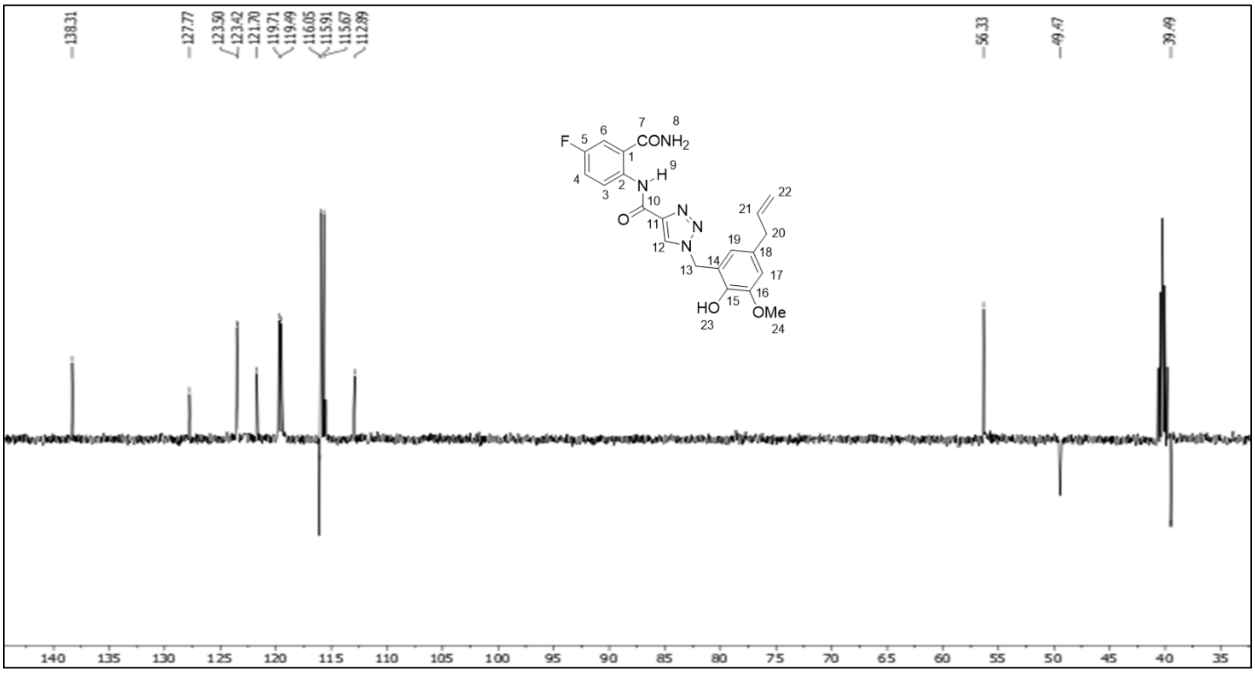

Figure S18. ESI-HRMS Spectrum for Compound **9c**

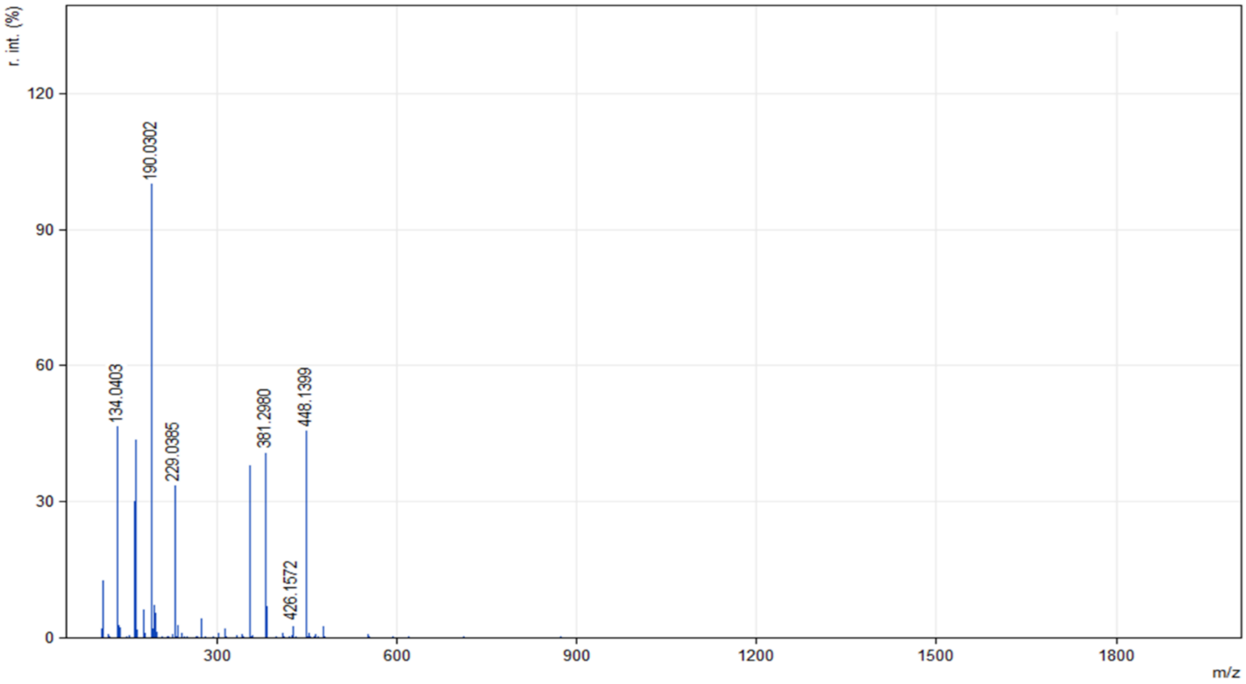

Figure S19. ^{1}H NMR Spectrum for Compound **9d** in DMSO-d_6 (400 MHz)

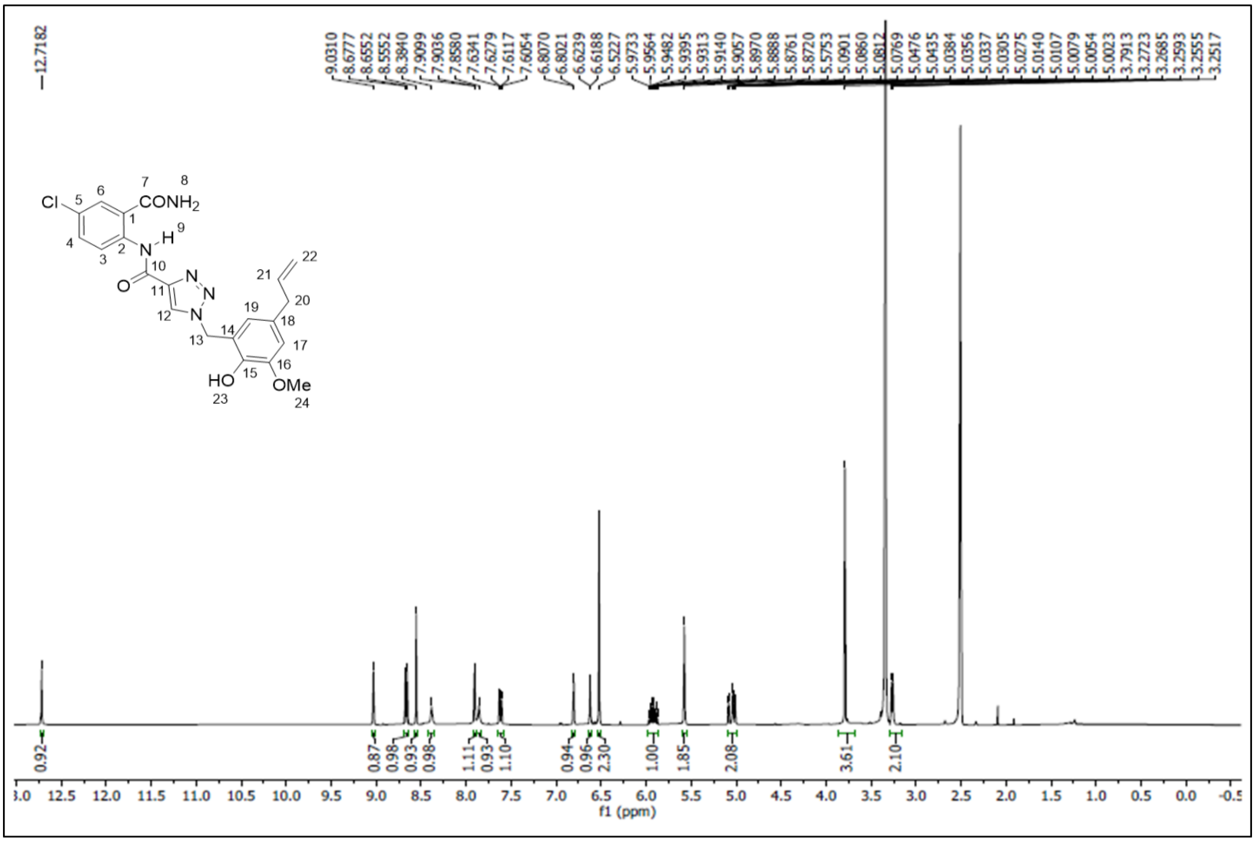

Figure S20. ^{13}C NMR Spectrum for Compound **9d** in DMSO-d_6 (100 MHz)

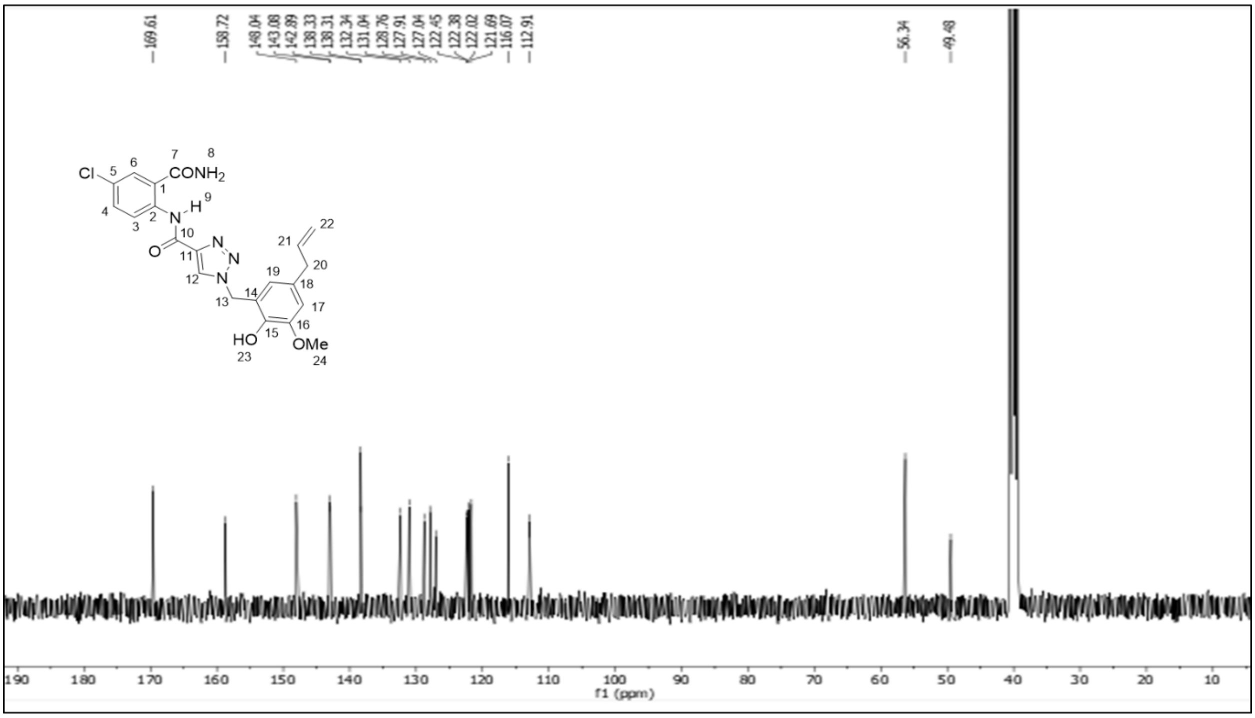

Figure S21. DEPT-135 Subspectrum for Compound **9d** in DMSO-d_6 (100 MHz)

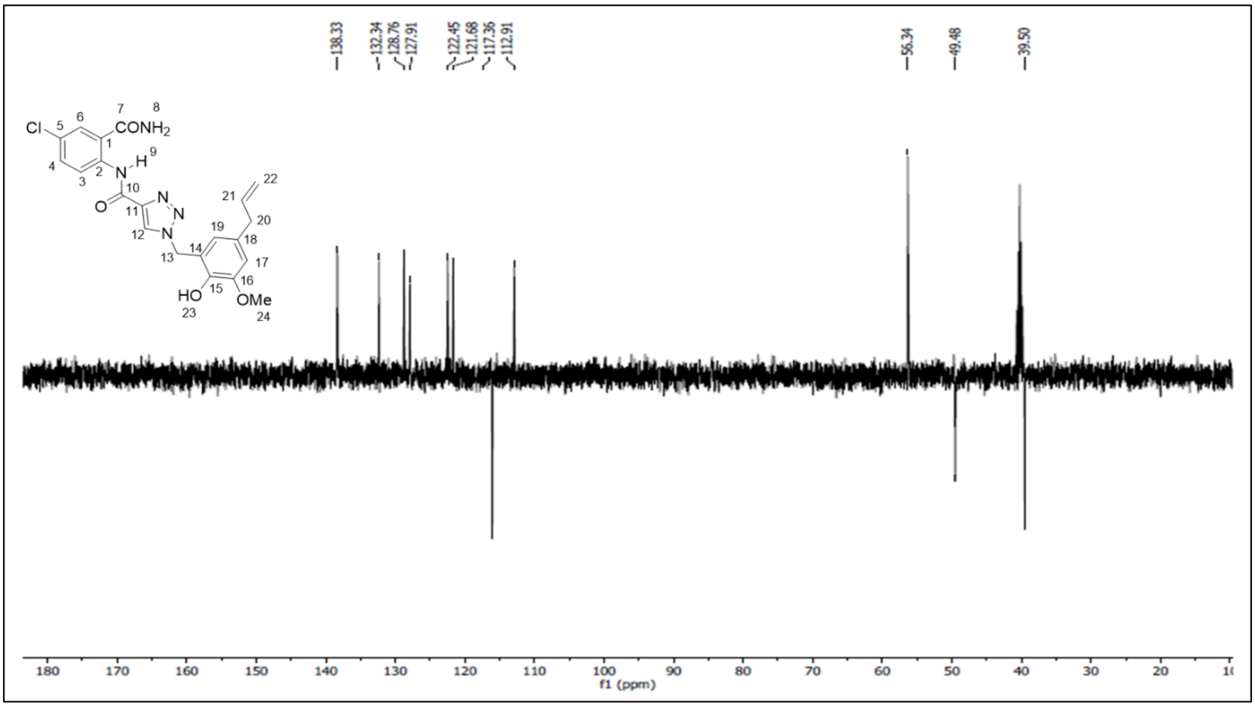

Figure S22. ESI-HRMS Spectrum for Compound **9d**

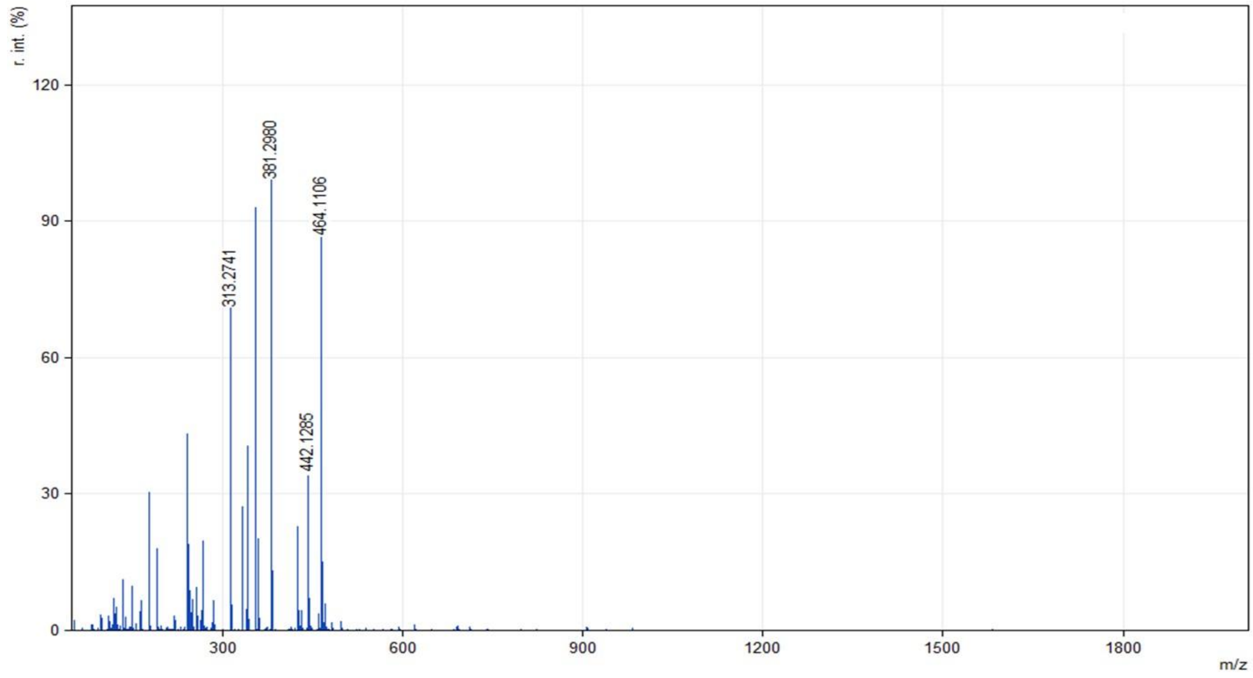

Figure S23. ^{1}H NMR Spectrum for Compound **10a** in DMSO-d_6 (400 MHz)

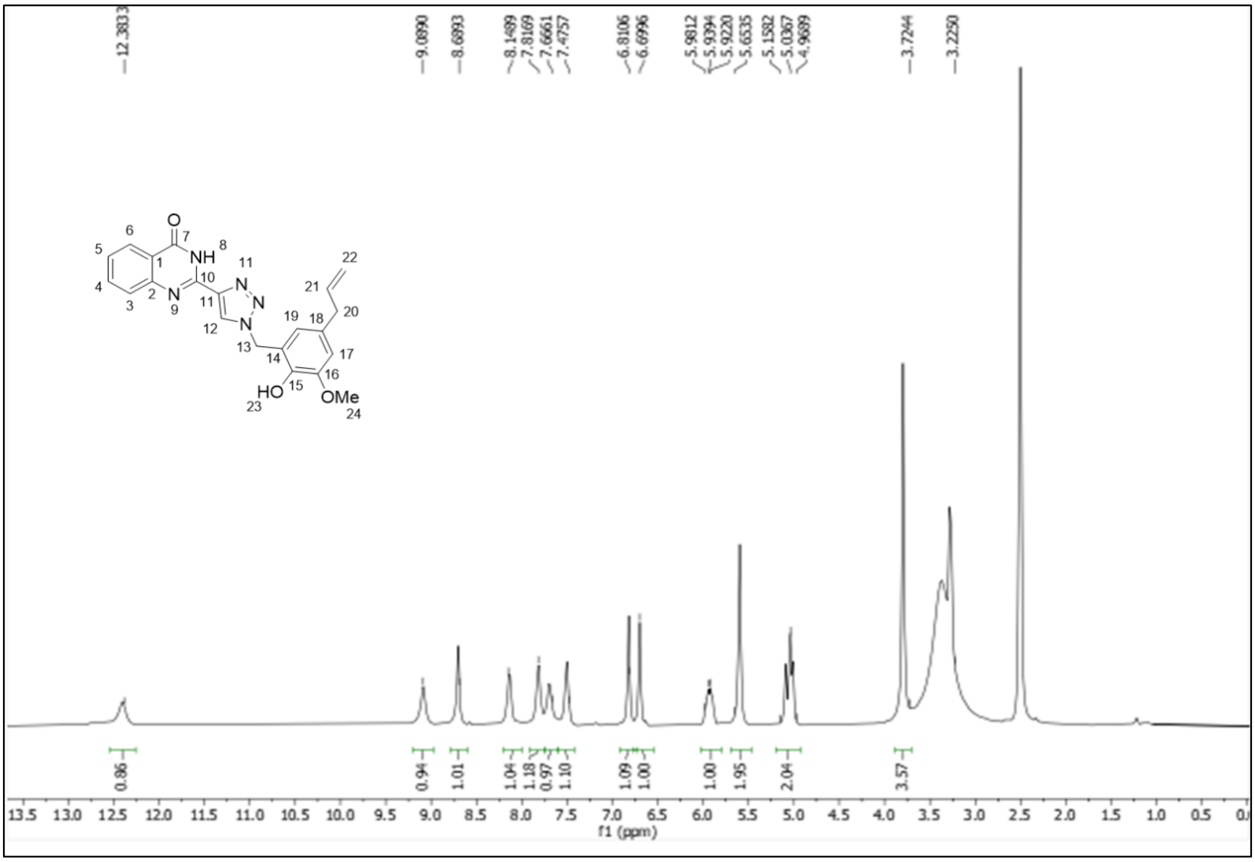

Figure S24. ^{13}C NMR Spectrum for Compound **10a** in DMSO-d_6 (100 MHz)

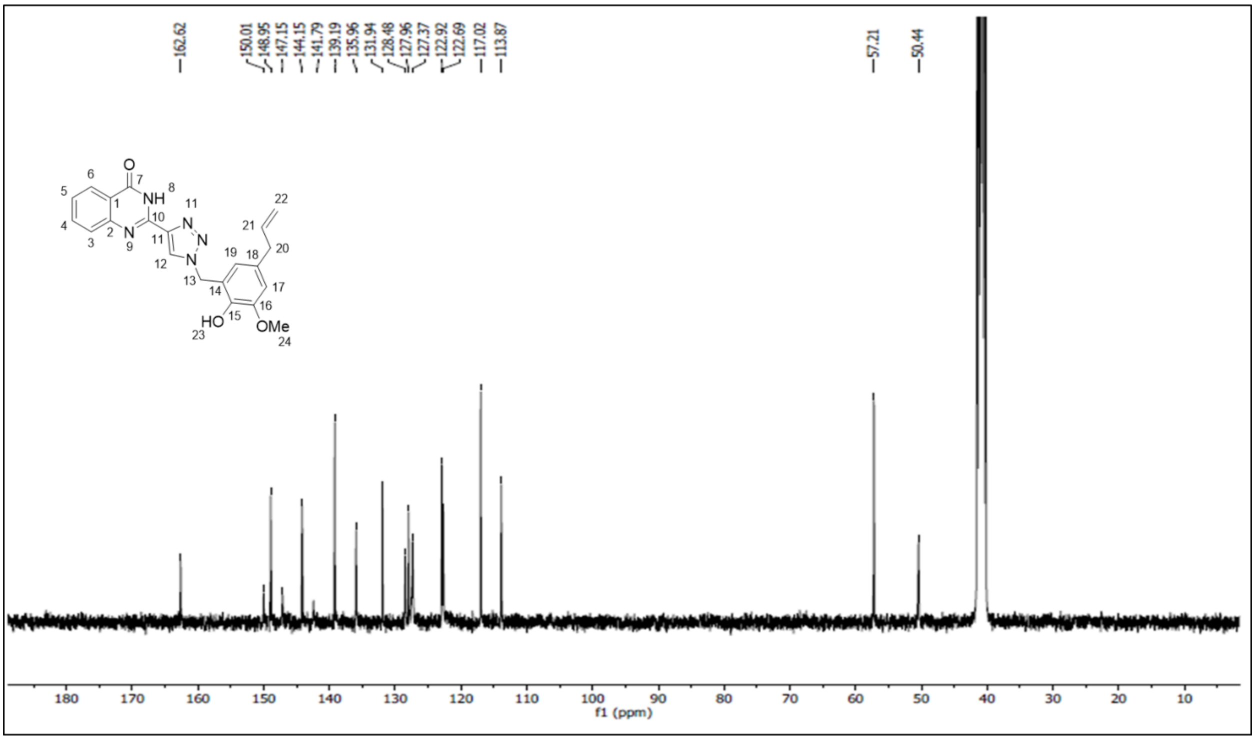

Figure S25. DEPT-135 Subspectrum for Compound **10a** in DMSO-d_6 (100 MHz)

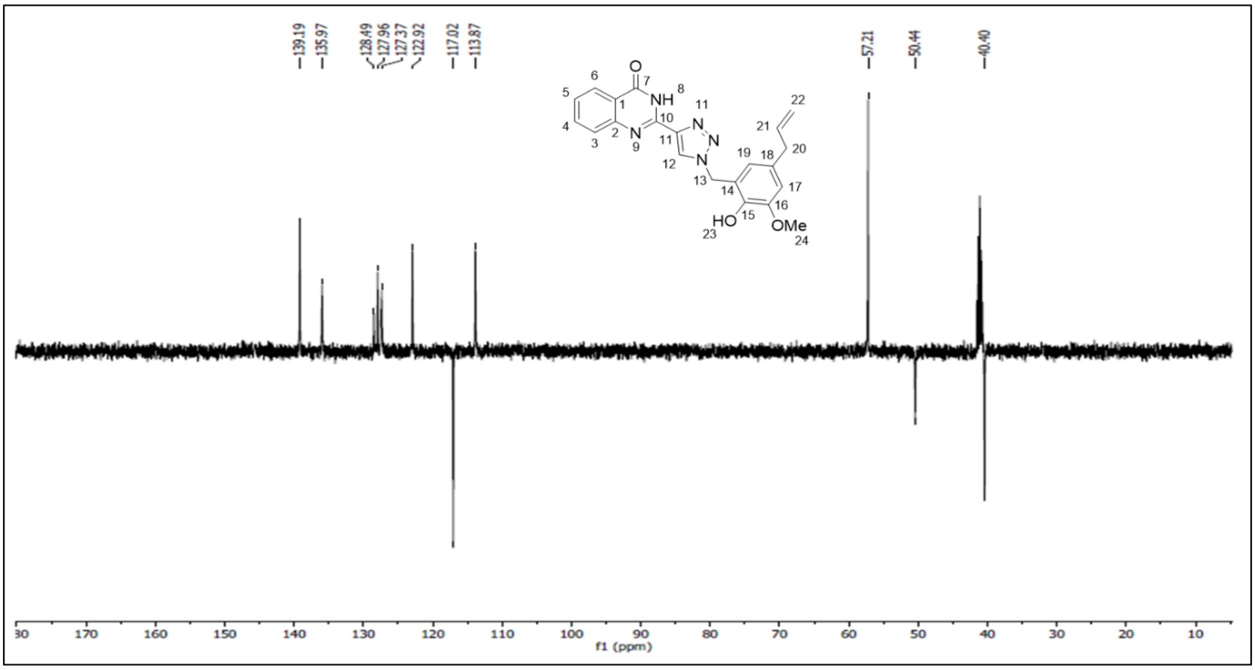

Figure S26. ESI-HRMS Spectrum for Compound **10a**

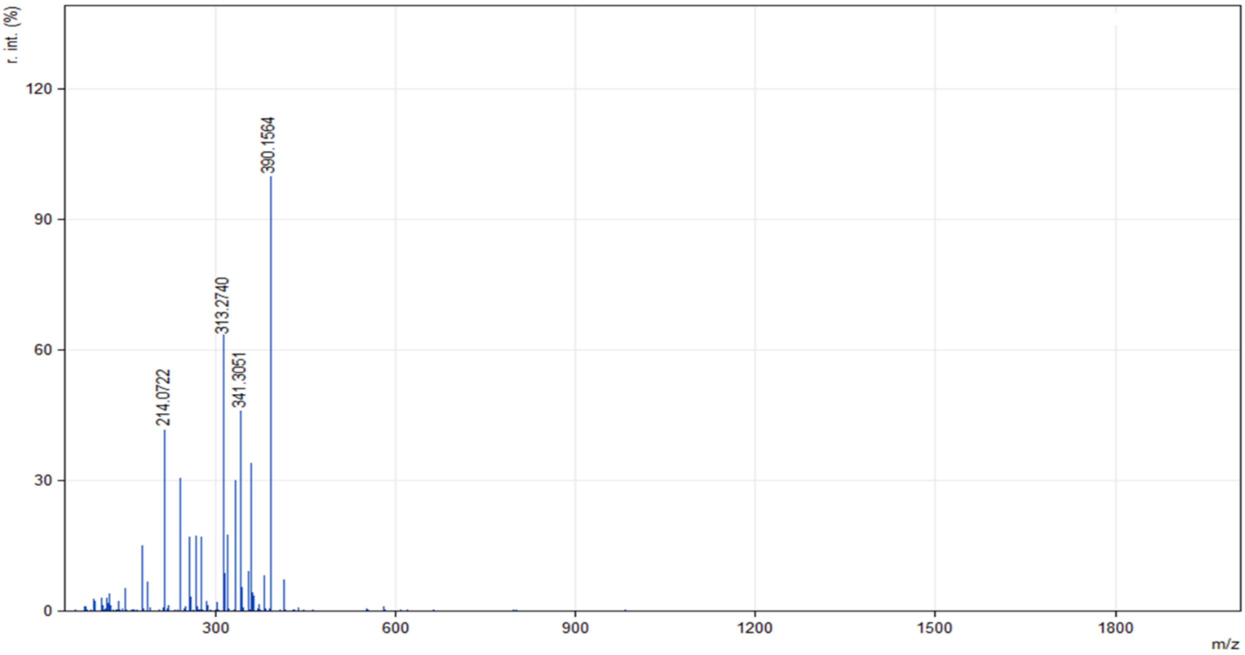

Figure S27. ^{1}H NMR Spectrum for Compound **10b** in DMSO-d_6 (400 MHz)

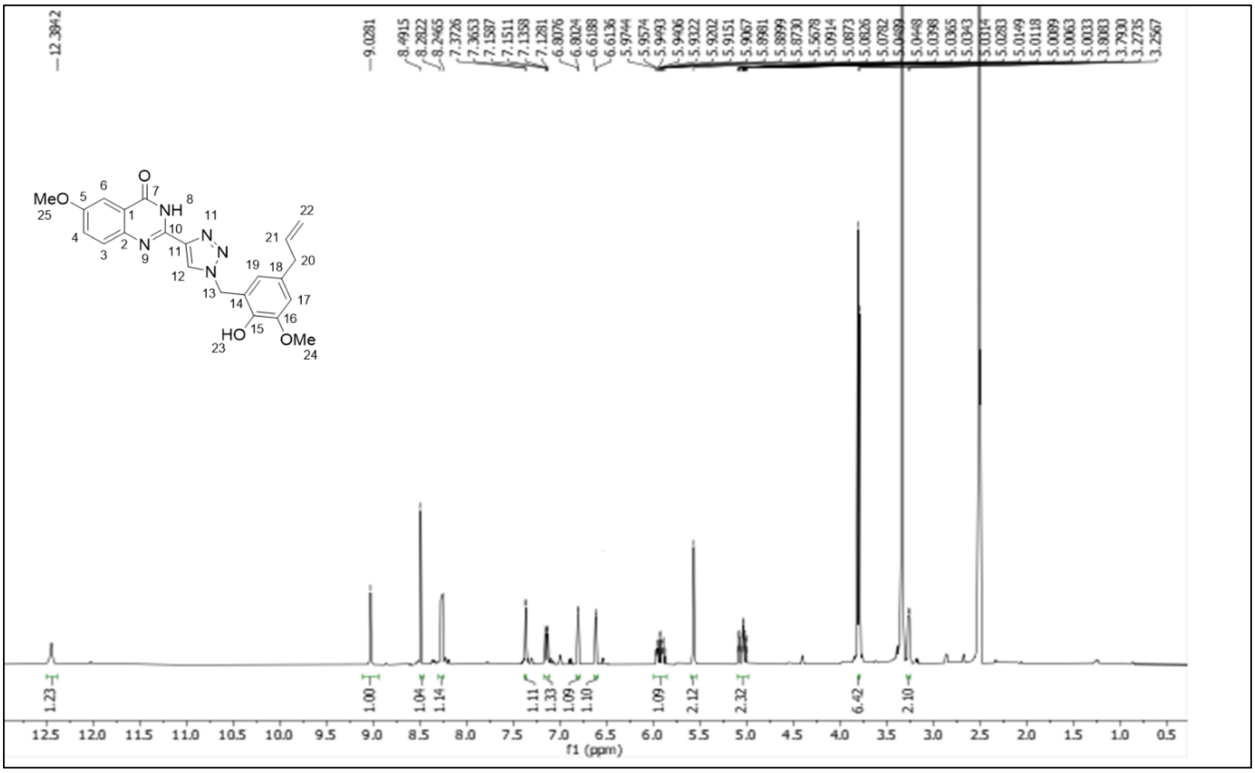

Figure S28. ^{13}C NMR Spectrum for Compound **10b** in DMSO-d_6 (100 MHz)

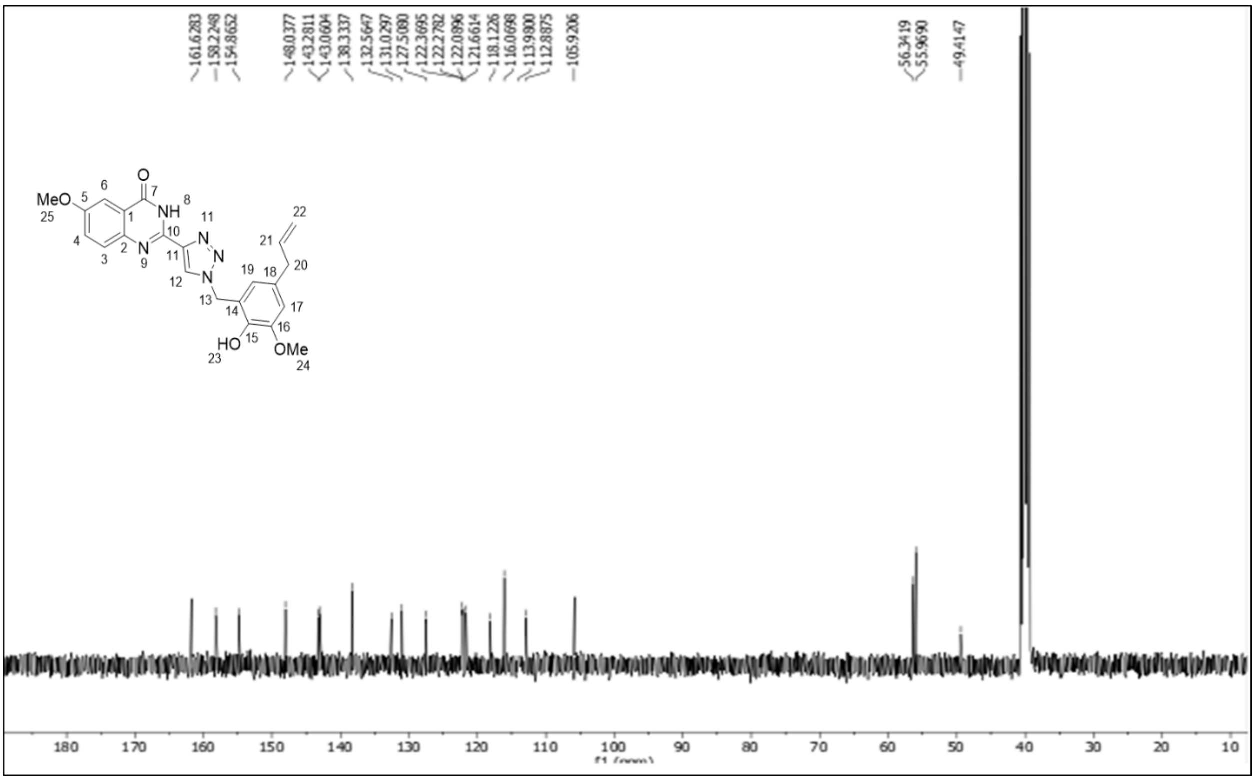

Figure S29. DEPT-135 Subspectrum for Compound **10b** in DMSO-d_6 (100 MHz)

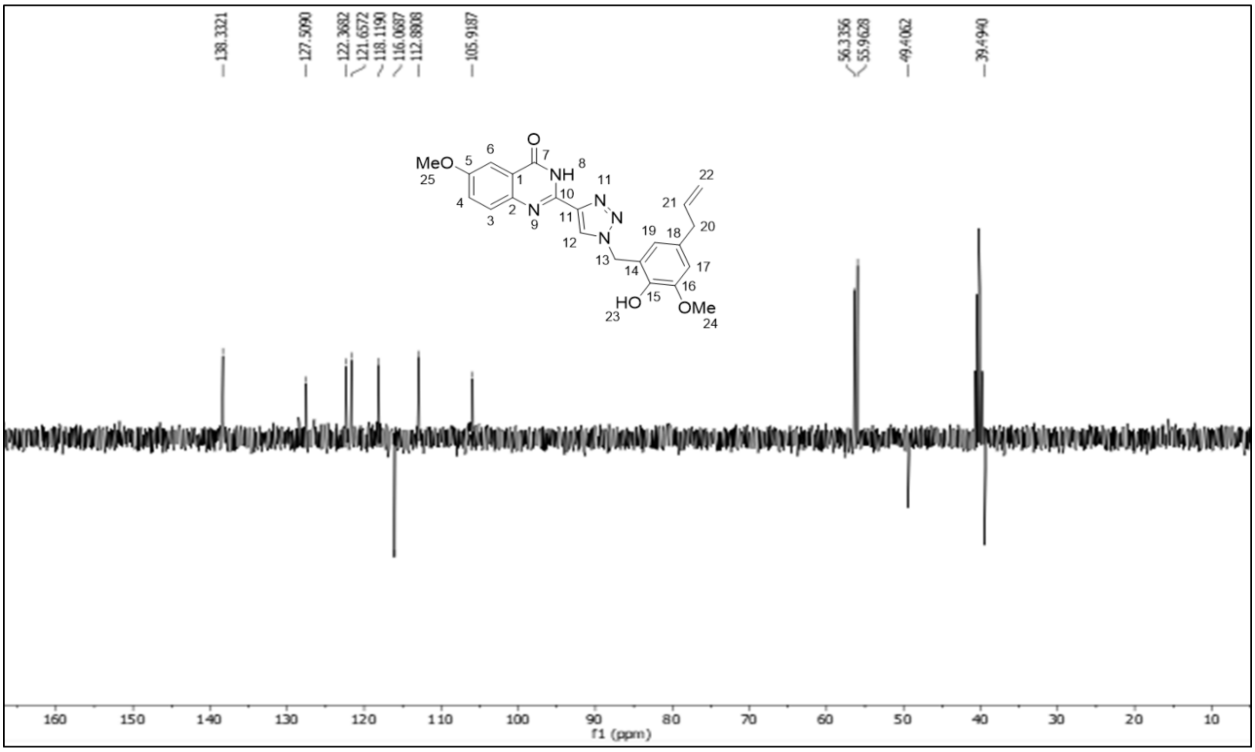

Figure S30. ESI-HRMS Spectrum for Compound **10b**

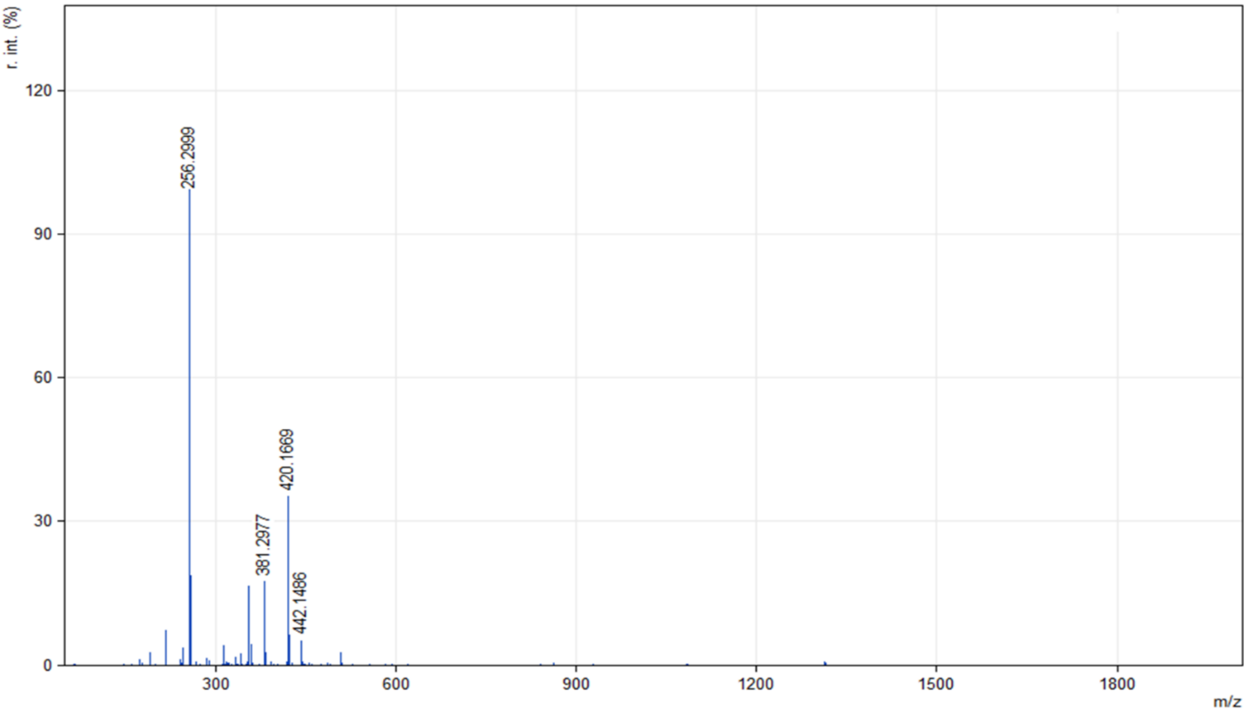

Figure S31. ^{1}H NMR Spectrum for Compound **10c** in DMSO-*d*$_6$ (400 MHz)

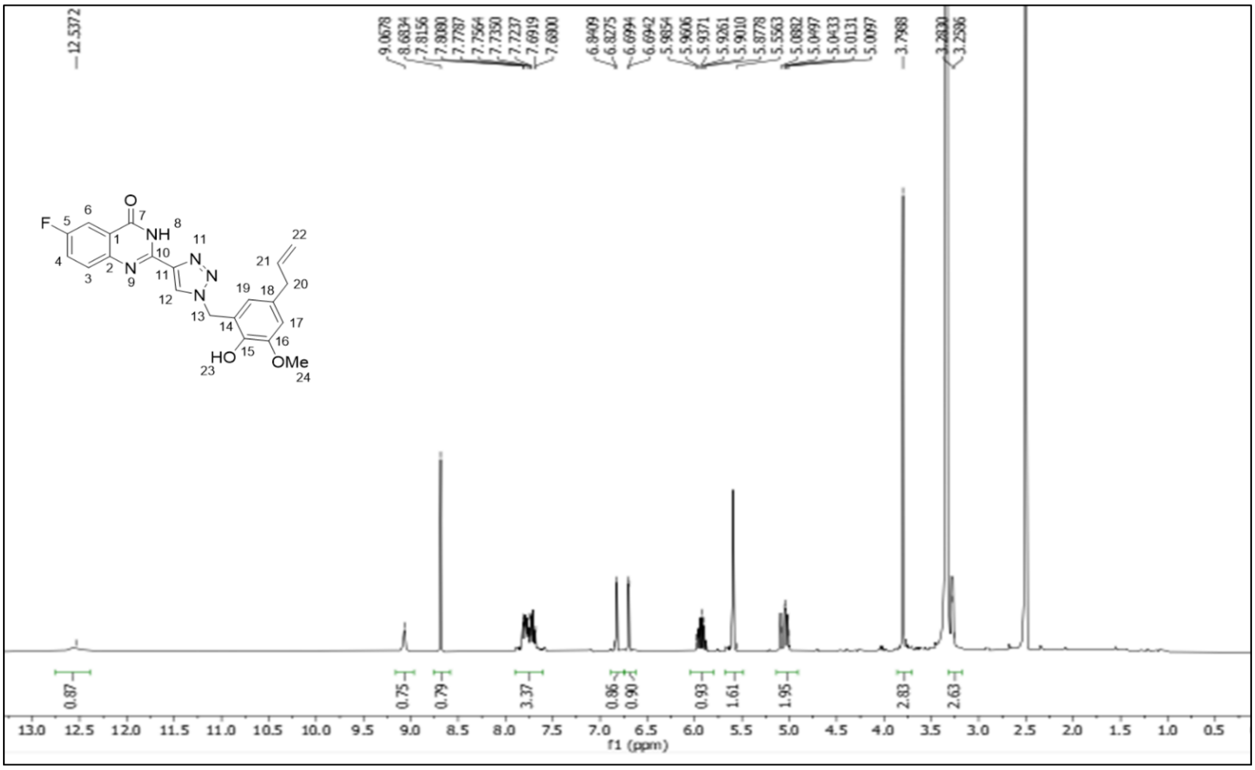

Figure S32. ^{13}C NMR Spectrum for Compound **10c** in DMSO-*d*$_6$ (100 MHz)

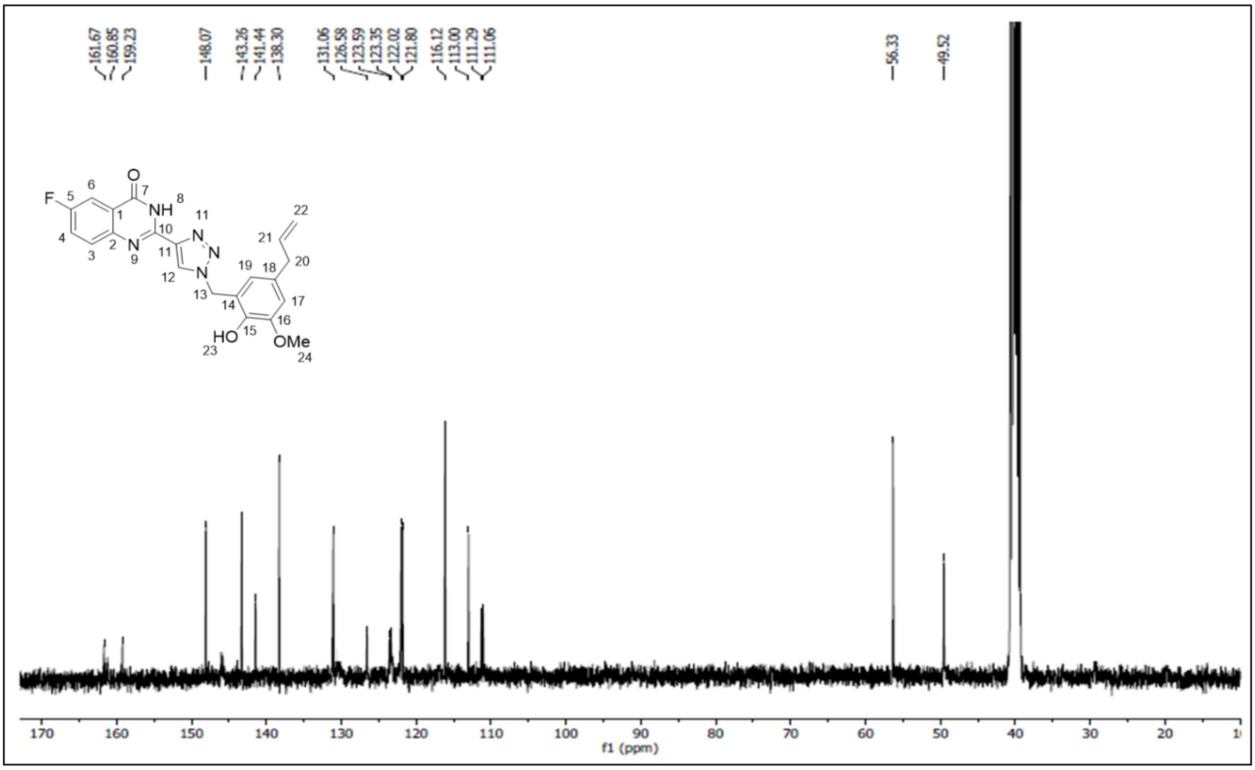

Figure S33. DEPT-135 Subspectrum for Compound **10c** in DMSO-d_6 (100 MHz)

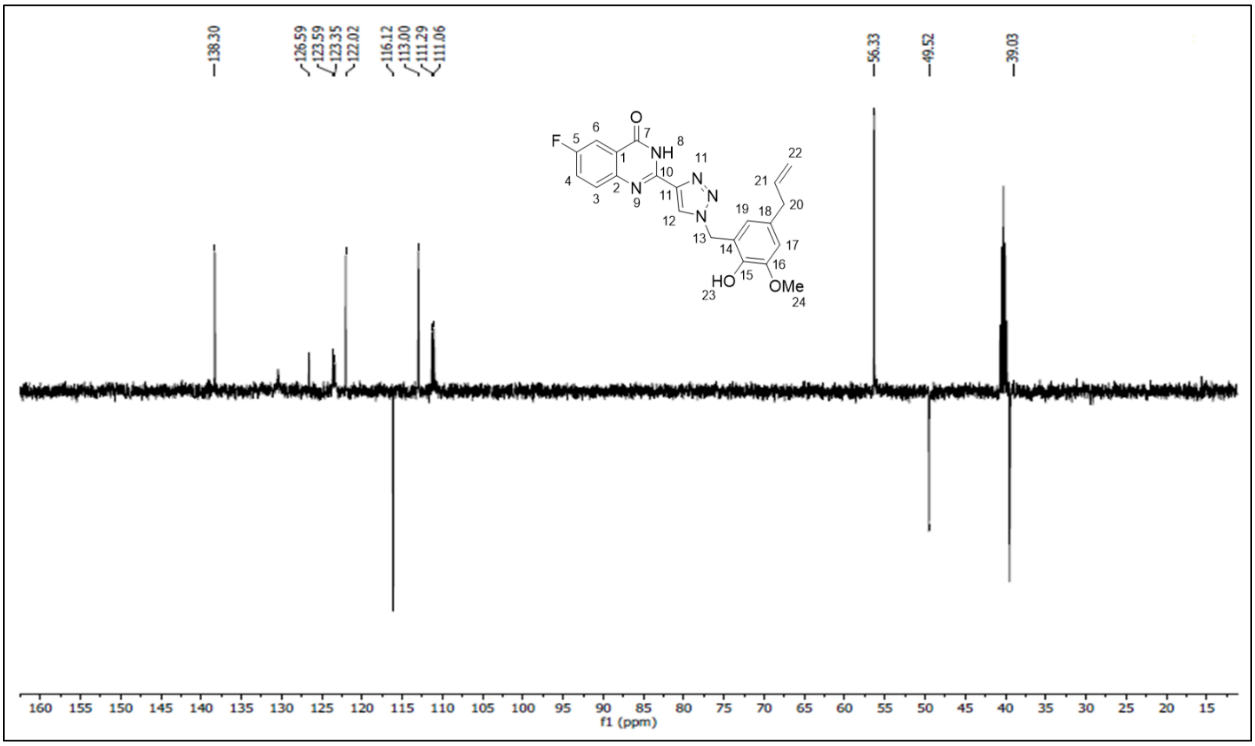

Figure S34. ESI-HRMS Spectrum for Compound **10c**

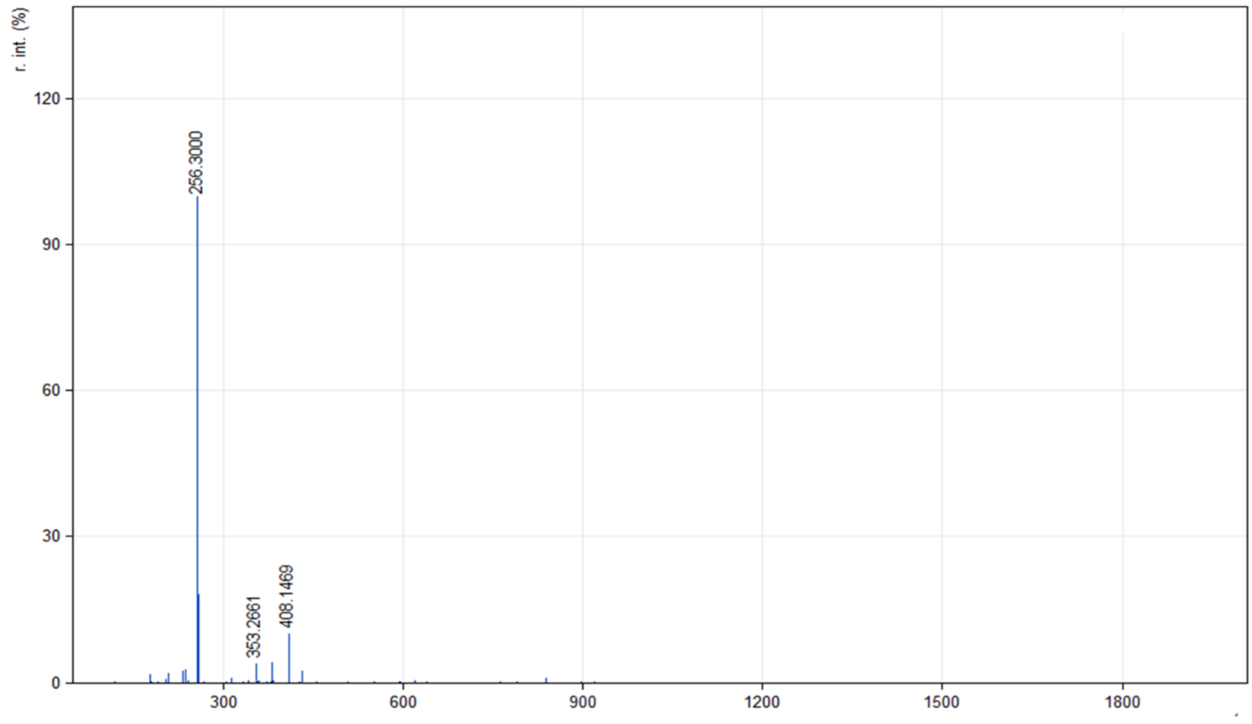

Figure S35. ^{1}H NMR Spectrum for Compound **13** in DMSO-d_6 (400 MHz)

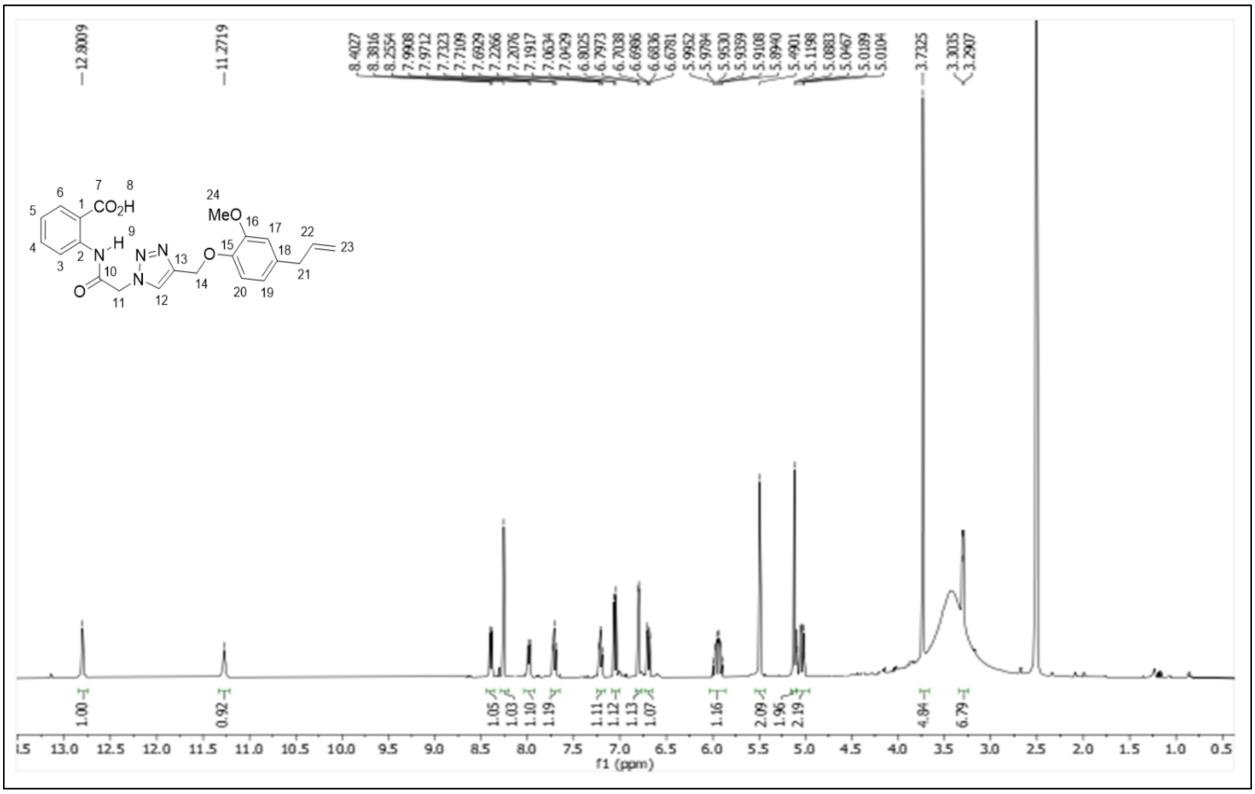

Figure S36. ^{13}C NMR Spectrum for Compound **13** in DMSO-d_6 (100 MHz)

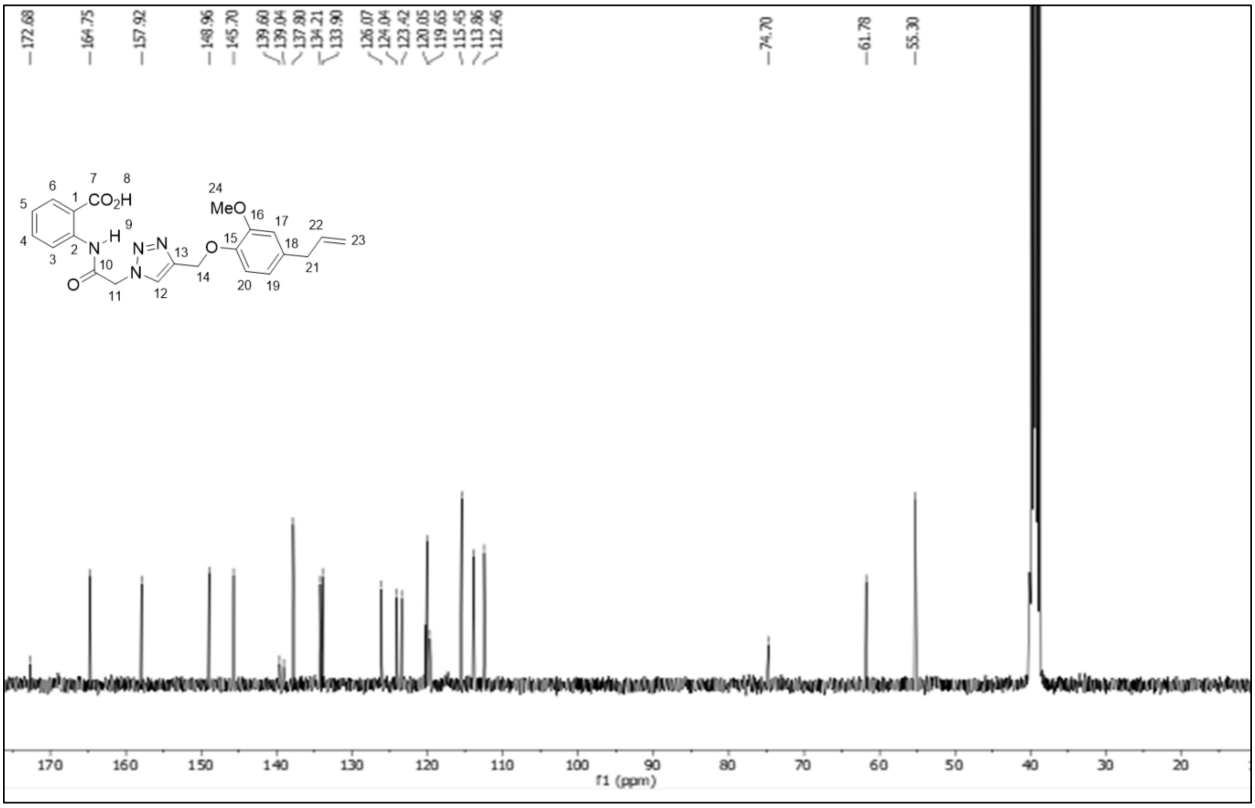

Figure S37. DEPT-135 Subspectrum for Compound **13** in DMSO-d_6 (100 MHz)

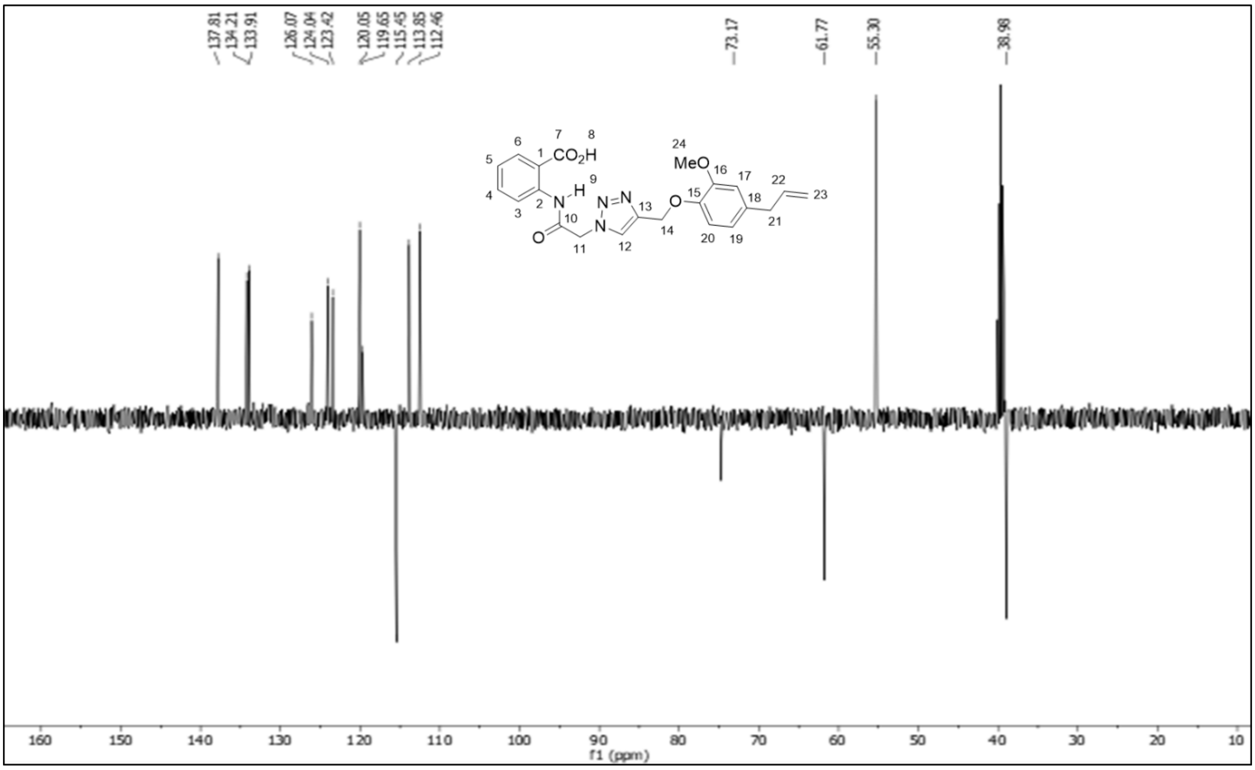

Figure S38. ESI-HRMS Spectrum for Compound **13**

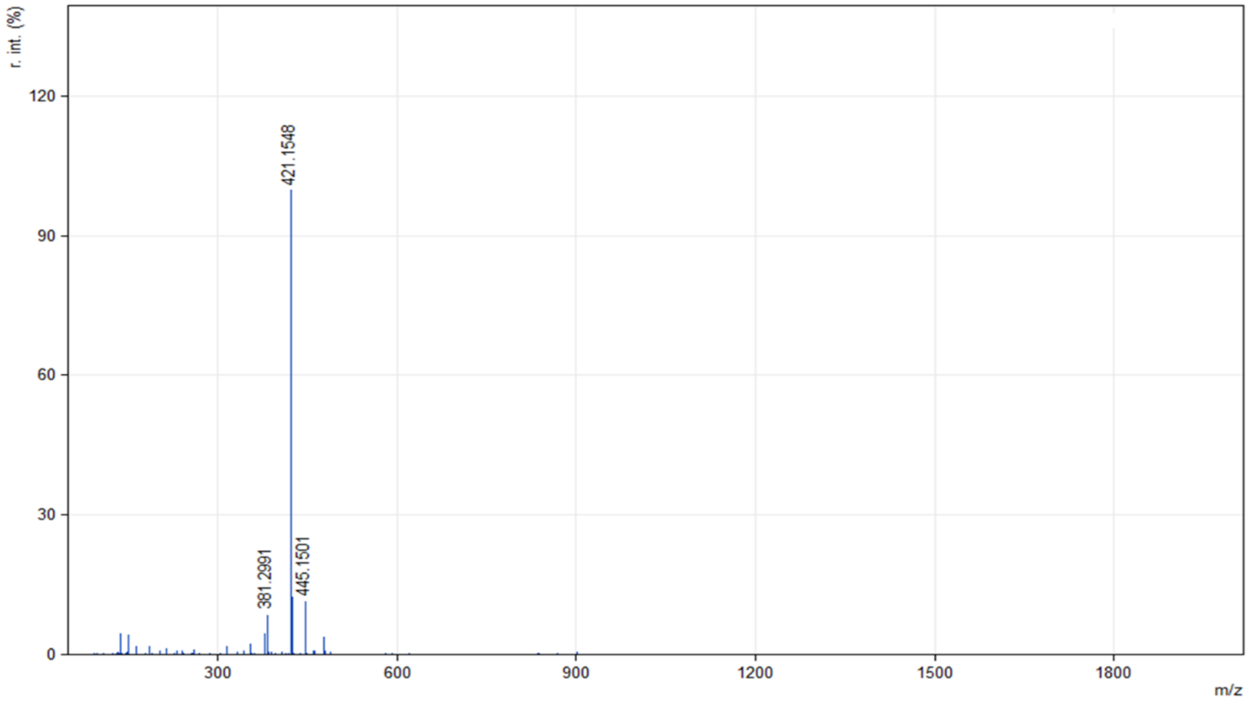

Figure S39. ^{1}H NMR Spectrum for Compound **14** in DMSO-d_6 (400 MHz)

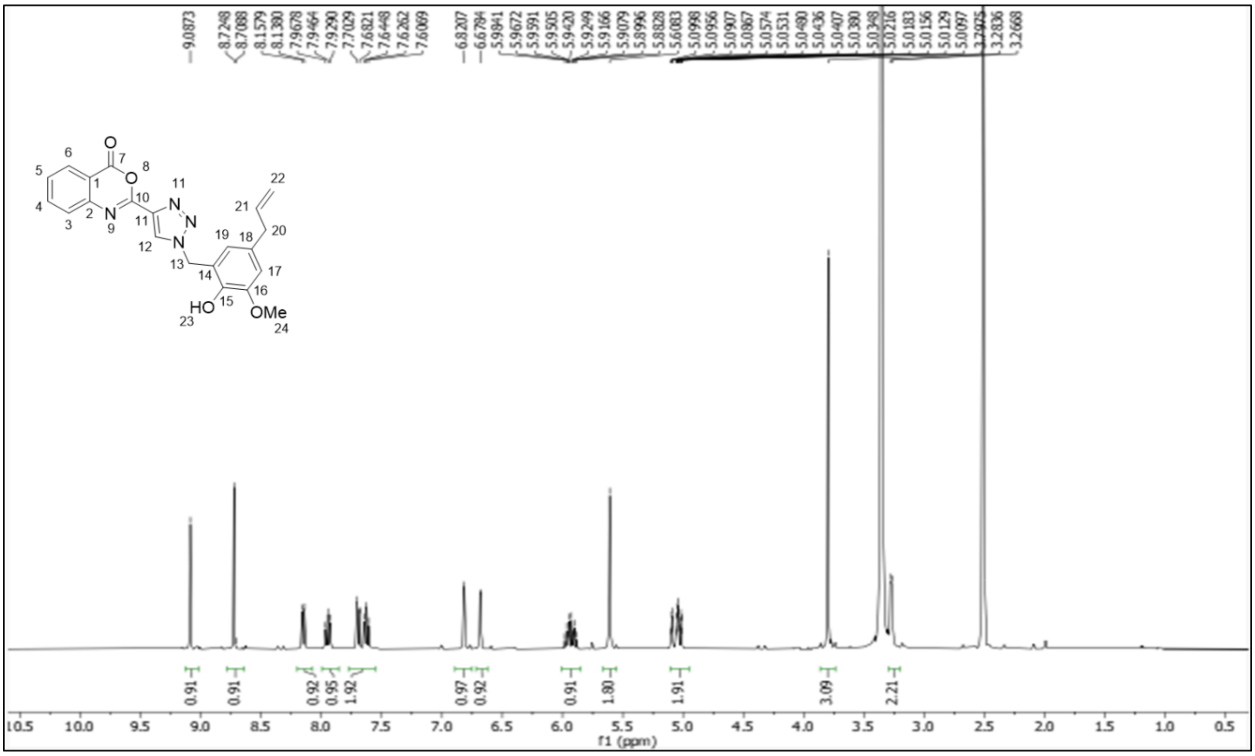

Figure S40. ^{13}C NMR Spectrum for Compound **14** in DMSO-d_6 (100 MHz)

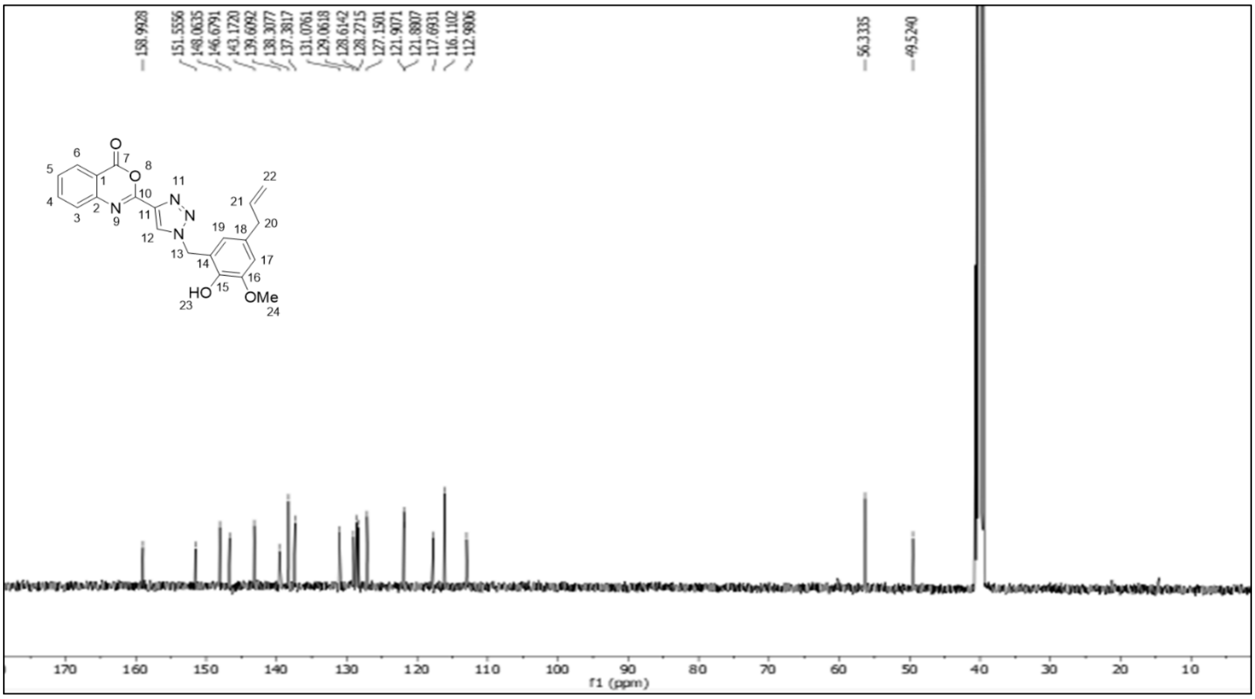

Figure S41. DEPT-135 Subspectrum for Compound **14** in DMSO-d_6 (100 MHz)

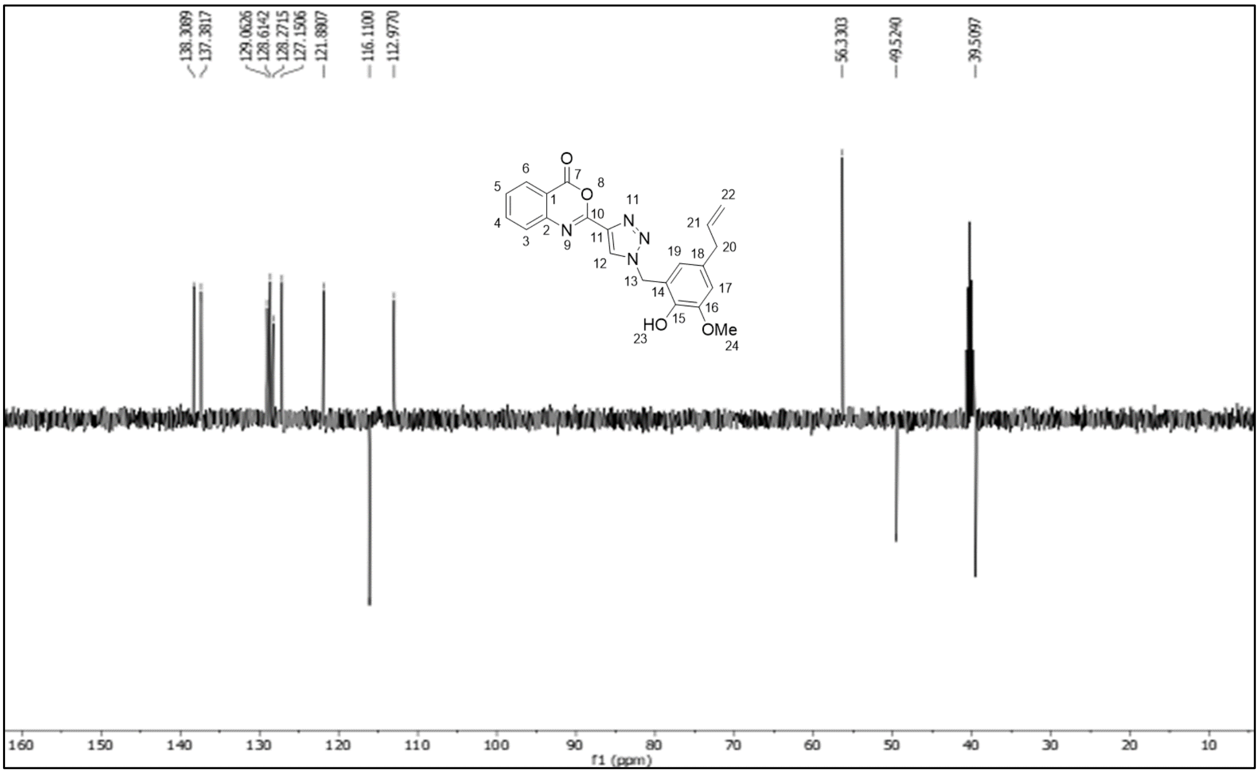

Figure S42. ESI-HRMS Spectrum for Compound **14**

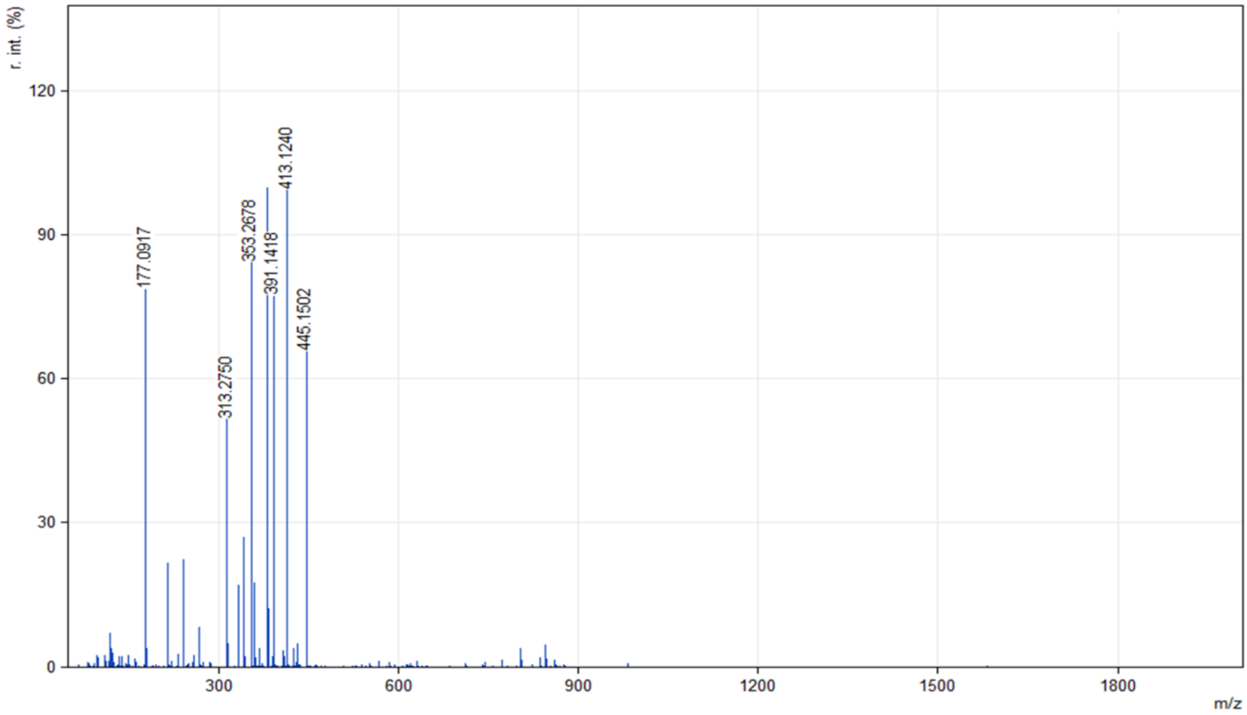

Table S1. Antimicrobial activity of compounds **9a**-**9d**, **10a**-**10c**, **13**, **14**, and **eugenol** against fungal strains: *Candida albicans*, *Aspergillus fumigatus* and *Trichophyton rubrum*, and against two bacteria strains: *Escherichia coli* and *Staphylococcus aureus*. The results of minimum inhibitory concentration (MIC) are expressed in µg/mL.

<table>
<tr><th>Strains/Compounds</th><th>C. albicans</th><th>T. rubrum</th><th>A. fumigatus</th><th>E. coli</th><th>S. aureus</th></tr>
<tr><td>9a</td><td rowspan="9">>512</td><td>512</td><td rowspan="9">>512</td><td rowspan="2">>512</td><td rowspan="2">>512</td></tr>
<tr><td>9b</td><td rowspan="2">>512</td></tr>
<tr><td>9c</td><td>256-512</td><td>256</td></tr>
<tr><td>9d</td><td rowspan="2">512</td><td rowspan="7">>512</td><td rowspan="7">>512</td></tr>
<tr><td>10a</td></tr>
<tr><td>10b</td><td>>512</td></tr>
<tr><td>10c</td><td>512</td></tr>
<tr><td>13</td><td>>512</td></tr>
<tr><td>14</td><td>256-512</td></tr>
<tr><td>Eugenol</td><td>512</td><td>256</td><td>512</td></tr>
</table>
